# Supplementary material for: Pulsatile inputs achieve tunable attenuation of gene expression variability and graded multi-gene regulation
Source: Nat Commun. 2018 Aug 30;9:3521. doi: 10.1038/s41467-018-05882-2 (PMC6117348; doi:10.1038/s41467-018-05882-2)
Supplement: Supplementary file 1 — Supplementary Information [file 41467_2018_5882_MOESM1_ESM.pdf]

Pulsatile inputs achieve tunable attenuation of gene expression variability and graded multi-gene regulation

Dirk Benzinger and Mustafa Khammash

Supplementary Information

Contains:
Supplementary Figures 1-14
Supplementary Notes 1-5
Supplementary Tables 1-7

Supplementary Figures:

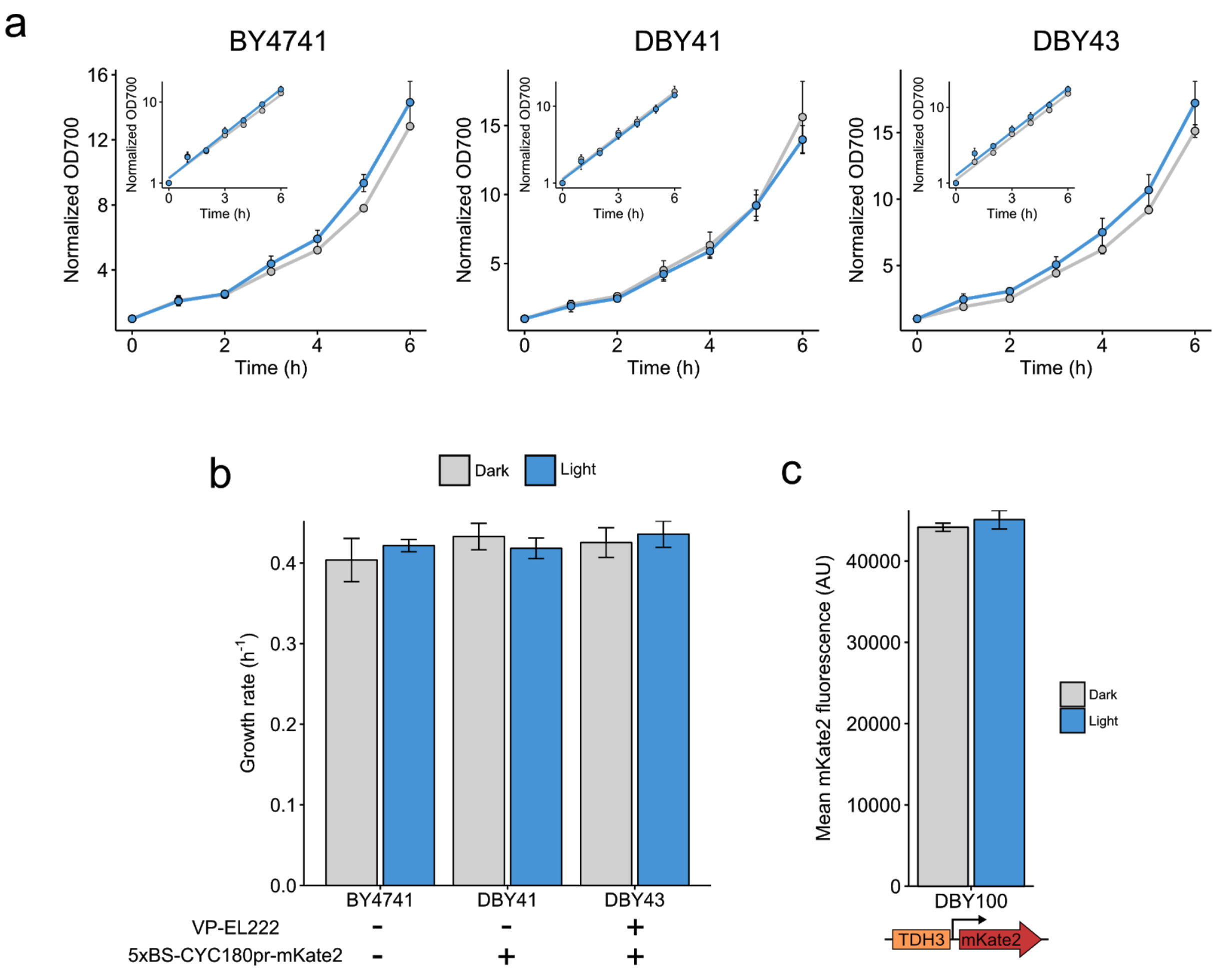

Supplementary Figure 1. VP-EL222 expression and illumination does not affect cell growth and constitutive gene expression. (**a**) Yeast strains, with or without the VP-EL222 and a reporter construct (5xBS-*CYC180*pr-mKate2), were grown in the dark (grey) or under blue light illumination (420 μW cm^{-2}) (blue) for 6 h. The OD_{700} was measured every hour and the growth rate was calculated by performing linear regressions on log-transformed OD-data (see inset). Data represents the mean and s.d. of two independent experiments and was normalized by the OD_{700} value at t=0. (**b**) Calculated growth rates for the growth experiments shown in (a). Data represents the mean and s.d. of two independent experiments. The average growth rate of all experiments is 0.42 h^{-1}. This value was used as the protein dilution rate in the mathematical modeling. (**c**) A yeast strain expressing mKate2 constitutively from the *TDH3* promoter (DBY100) was grown either in the dark or under illumination (420 μW cm^{-2}) for 6 h. The average cellular mKate2 fluorescence was measured using flow cytometry. Data represents the mean and s.d. of two independent experiments.

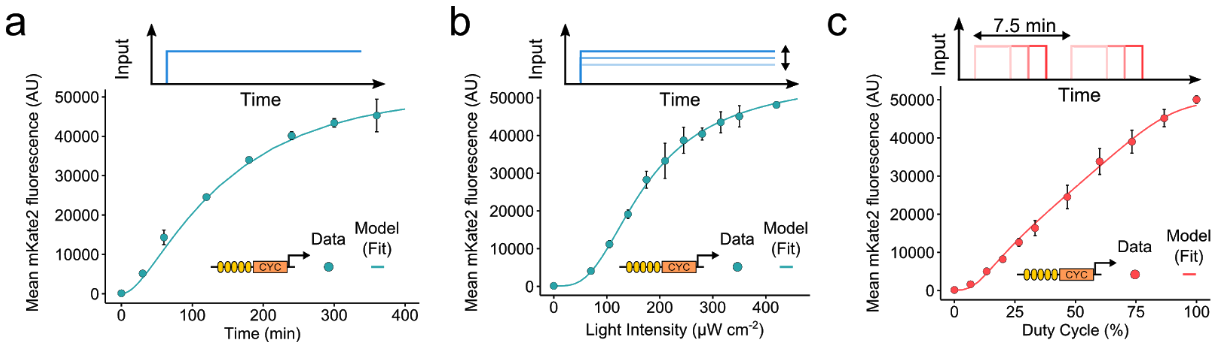

Supplementary Figure 2. Characterization experiments and results of model fitting. Three characterization experiments were performed with DBY43 (containing the VP-EL222 and a reporter construct (5xBS-*CYC180*pr-mKate2)). Data points represent mean cellular mKate2 fluorescence (measured by flow cytometry, mean and s.d. of three independent experiments) and lines represent the model fits. (**a**) Time-course of VP-EL222 mediated gene expression. Cells were illuminated with blue light (350 μW cm^{-2}) for 6 h and fluorescence was measured at regular intervals. (**b**) Dose response to AM / light intensity. Fluorescence was measured after 6 h of illumination. (**c**) Dose response to PWM with a 7.5 min period. The light intensity was 420 μW cm^{-2}.

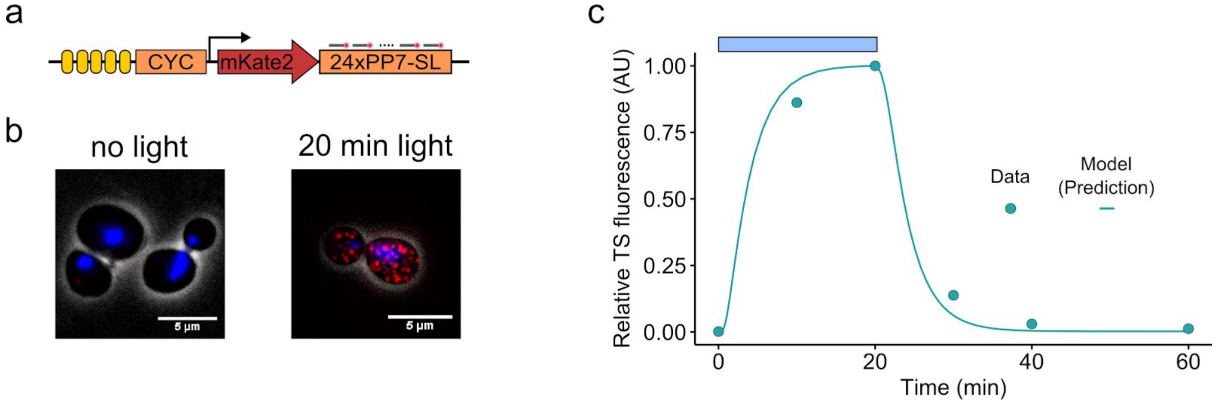

Supplementary Figure 3. smFISH analysis of VP-EL222 mediated transcription. A yeast strain containing VP-EL222 and a reporter construct containing a sequence coding for 24 copies of the PP7 stem loop (5xBS-*CYC180*pr-mKate2-24xPP7-SL) were grown under blue light illumination (420 μW cm^{-2}) for 20 min and subsequently in the dark for 40 min. Samples were taken before illumination and after 10, 20, 30, 40, 60 min. smFISH was performed with CY3 labeled probes complementary to the PP7-SL (probe sequences are listed in **Supplementary Table 3**). (**a**) Schematic of the reporter construct. (**b**) Representative microscopy images before and after 20 min of illumination. Grayscale: phase contrast / cell boundaries, blue: DAPI channel (maximum intensity projection), red: CY3 channel (maximum intensity projection). Scale bars represents 5 μm (**c**) Time-course of nascent RNA quantification. Points represent measured fluorescence at the transcription site (TS) relative to the maximal value at 20 min (see **Methods** for details on the quantification). The line represents the model prediction for nascent RNA accumulation (see **Supplementary Note 1 'Estimating nascent RNA accumulation'** for modeling details).

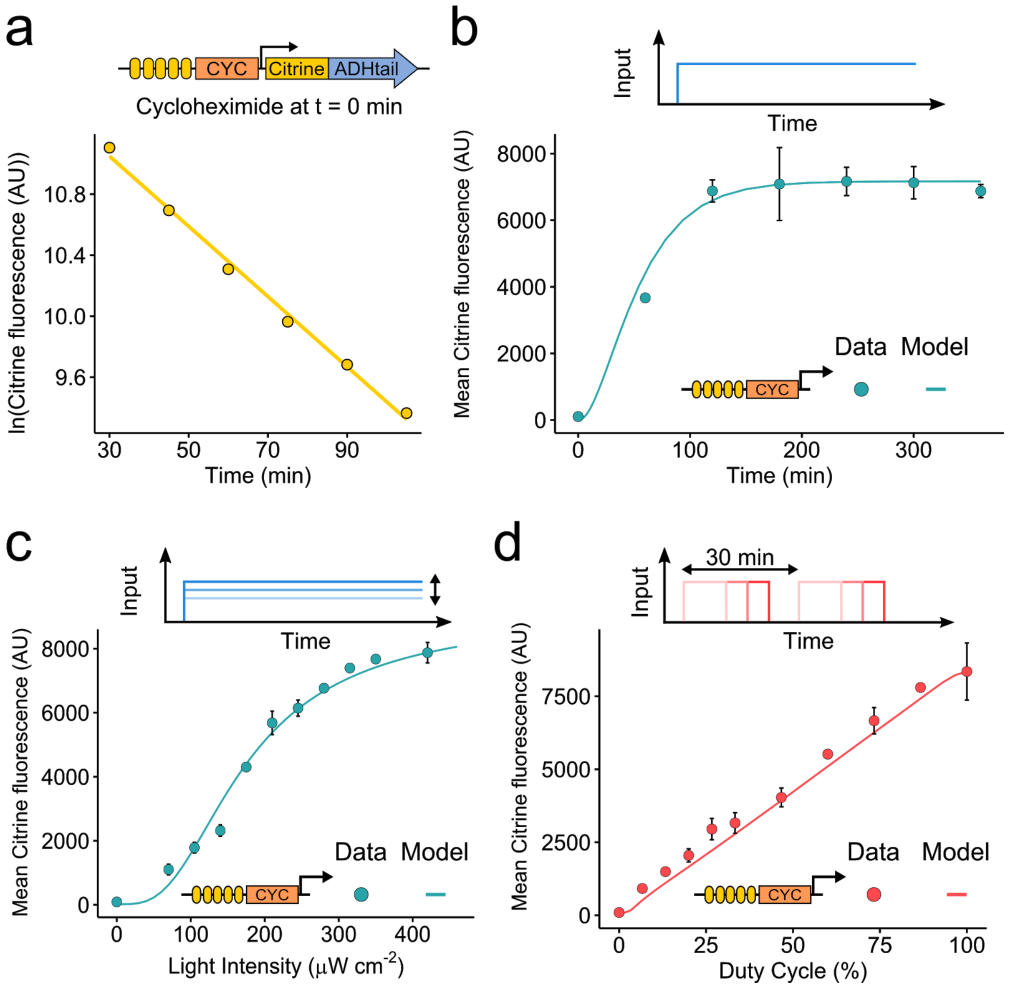

Supplementary Figure 4. Characterization experiments of destabilized FP expression. Four characterization experiments were performed with DBY149, expressing the Citrine-*ADH*tail FP [1,2] construct under control of 5xBS-*CYC180*pr. (**a**) Degradation rate measurement for the destabilized Citrine FP variant. DBY149 was illuminated with blue light (420 µW cm^{-2}) for 6 h after which cycloheximide was added to the culture to a final concentration of 0.1 mg/ml. Fluorescence was measured every 15 min starting 30 min after cycloheximide addition. The degradation rate was found to be 0.023 min^{-1} based on a linear regression to log-transformed fluorescence data (see graph). (**b**) Time-course of VP-EL222 mediated gene expression. Cells were illuminated with blue light (350 µW cm^{-2}) for 6 h and fluorescence was measured at regular intervals. (**c**) Dose response to AM / light intensity. Fluorescence was measured after 6 h of illumination. (**d**) Dose response to PWM with a 30 min period. The light intensity was 420 µW cm^{-2}. For (b)-(d) data points represent mean cellular Citrine fluorescence (mean and s.d. of two independent experiments) and lines represent the modeling results. Model parameters were the same as used for the stable FP mKate2 (see **Supplementary Table 4**) but the protein degradation rate k_{degP} was adjusted to 0.03 min^{-1} (pure protein degradation rate (see (a)) + dilution by cell growth). Model predictions were multiplied by a constant to bring experimental and modeling results to the same maximal value. This normalization was necessary as characterization experiments for model fits were performed solely based on experiments using the stable mKate2 fluorescent protein as a reporter.

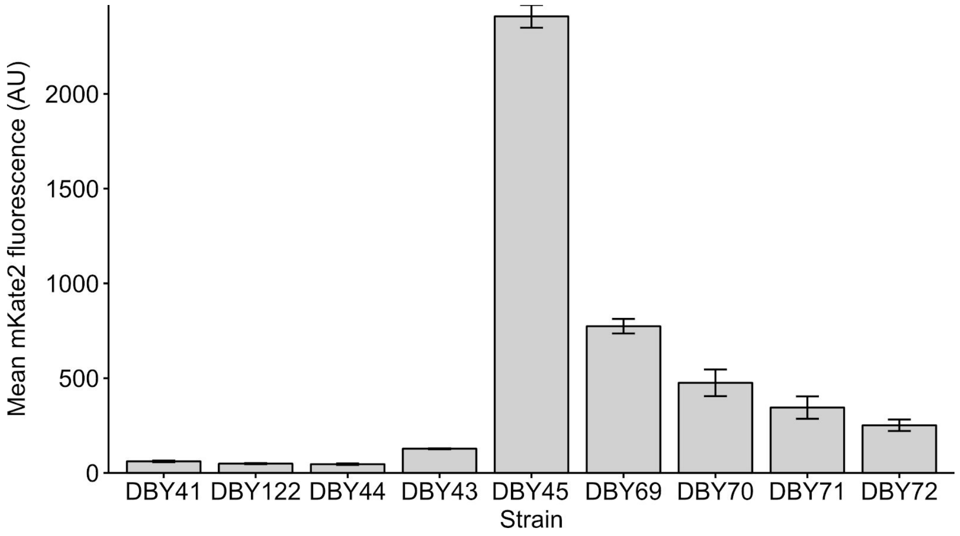

Supplementary Figure 5. Comparison of expression from different VP-EL222 regulated promoters in the dark. Strains, expressing mKate2 under the control of different promoters (see **Supplementary Table 2**), were cultured for 6h in the dark. The average cellular mKate2 fluorescence was measured using flow cytometry. Data represents the mean and s.d. of three independent experiments.

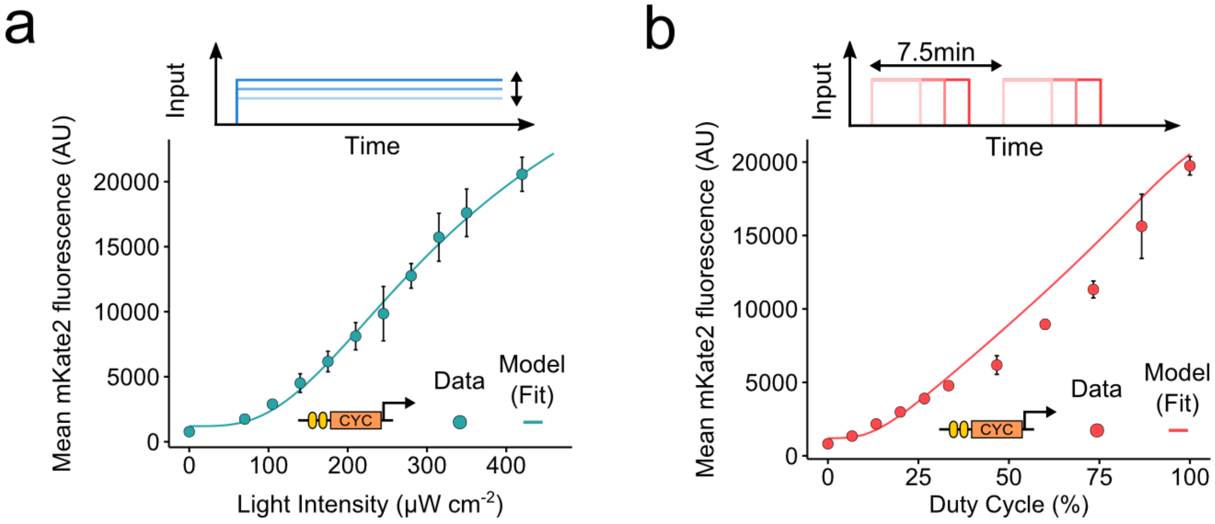

Supplementary Figure 6. Model fits for a VP-EL222 regulated promoter based on the *CYC180* promoter with two VP-EL222 binding sites (2xBS-*CYC180*pr, DBY69). Data points represent mean cellular mKate2 fluorescence (measured by flow cytometry after 6 h of illumination, mean and s.d. of three independent experiments) and lines represent the model fits. (**a**) Dose response to AM / light intensity. (**b**) Dose response to PWM / duty cycle. The PWM period was 7.5 min. The light intensity was 420 μW cm^{-2}. See also **Supplementary Note 1 'Refitting of promoter-specific model parameters'**.

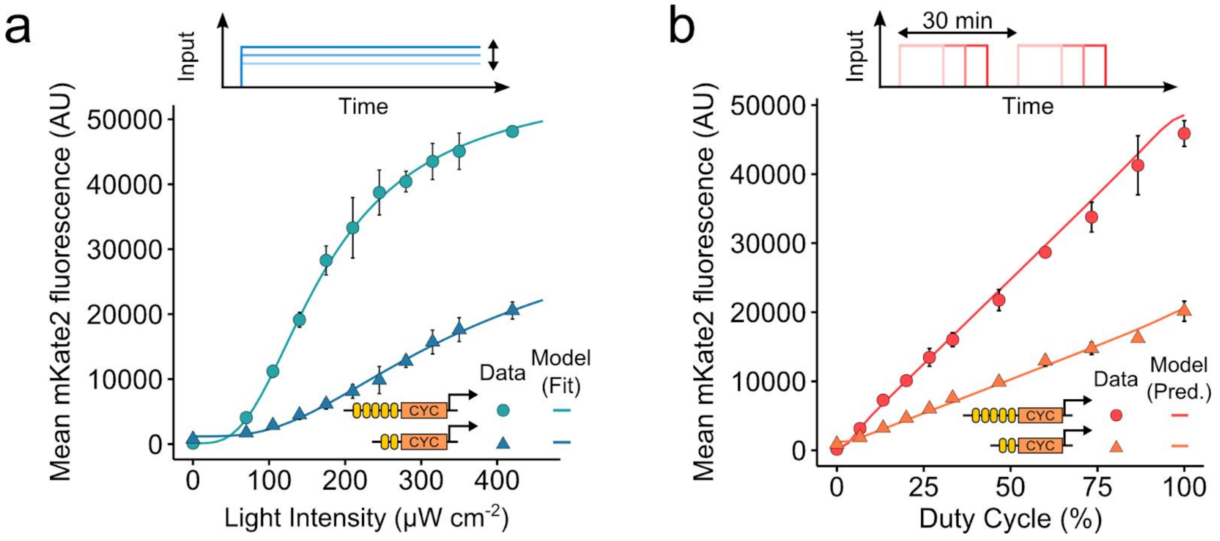

Supplementary Figure 7. Non-normalized curves showing the coregulation of 2xBS-*CYC180*pr and 5xBS-*CYC180*pr. Dose-response of the two promoters to AM (**a**) and PWM (**b**). Strains expressing mKate2 under the control of either a *CYC180* promoter with five (circle, 5xBS) or two (triangle, 2xBS) VP-EL222 binding sites were grown under the illumination conditions depicted on the x-axis for 6 h. The light intensity and period for PWM were 420 μW cm^{-2} and 30 min. Data represents the mean and s.d. of three independent experiments. Lines represent model fits or predictions.

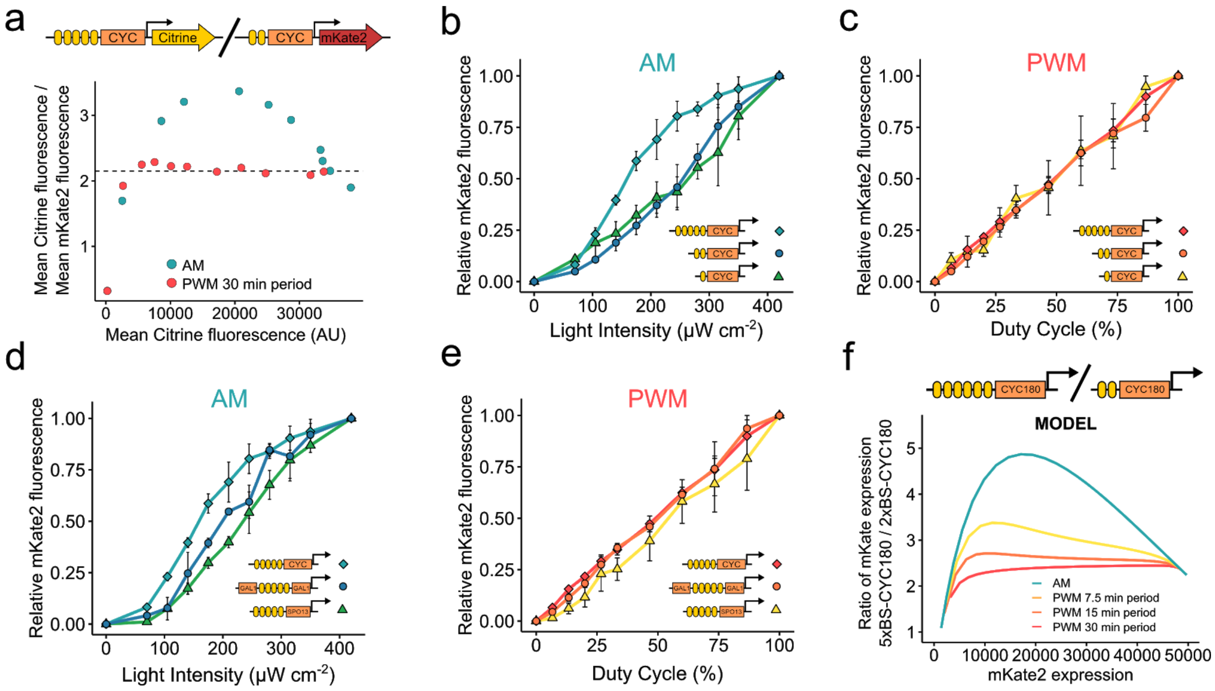

Supplementary Figure 8. Further results on PWM mediated co-regulation. (**a**) Co-regulation of 5xBS-*CYC180*pr-mCitrine and 2xBS-*CYC180*pr-mKate2 in single cells. The diploid yeast strain DBY118 was grown under different induction conditions for 6h and fluorescence was analyzed using flow-cytometry. PWM was performed with a 30 min period and a light intensity of 420 μW cm^{-2}. AM was performed with the following light intensities: 105, 140, 175, 210, 245, 280, 315, 350, 420, 490 μW cm^{-2}. The dashed line represents this ratio at constant illumination with a light intensity of 420 μW cm^{-2}. **(b) - (e)** Dose response of promoters differing in their binding site numbers (b,c) or promoter backbones (d,e) to AM (b,d) and PWM (c,e). Strains expressing mKate2 under the control of the respective promoters (depicted schematically on the plots) were grown under the illumination conditions depicted on the x-axis for 6 h. The light intensity and period for PWM were 420 μW cm^{-2} and 30 min. Mean cellular fluorescence measurements were normalized to be 0 in the dark and 1 at the highest input level to allow for easy comparison. Data represents the mean and s.d. of two to three independent experiments. (**f**) Model results for the effect of AM and PWM with different periods on co-regulation of gene expression from 5xBS-*CYC180*pr and 2xBS-*CYC180*pr.

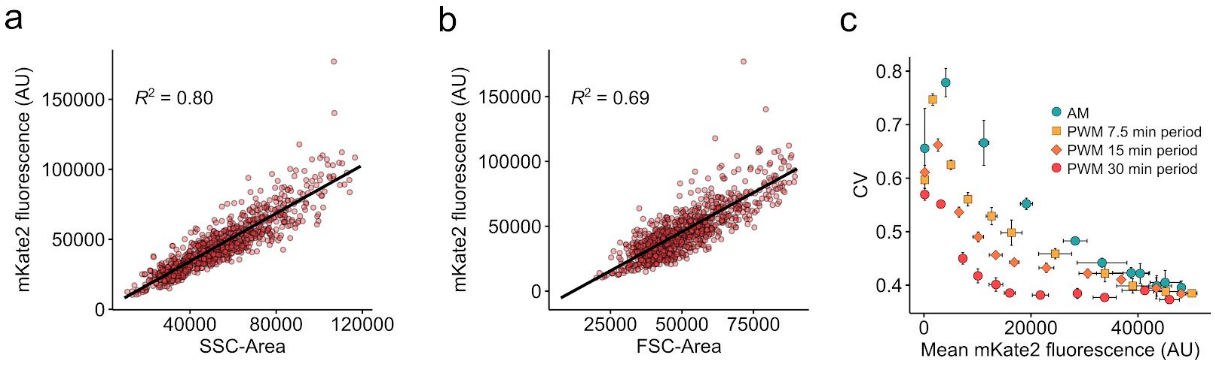

Supplementary Figure 9. Normalization of cellular fluorescence by scatter as a proxy for cell size. Differences in cell size can lead to strong cell-to-cell variability in cellular protein abundances that can mask the magnitude of other variability sources. We thus analyzed the correlation of side scatter (**a**, SSC) and forward scatter (**b**, FSC) with constitutive mKate2 expression from the *TDH3* promoter. Points represent individual cells. Lines represent linear regressions with the R^2 values shown on the graph. We found that both SSC and FSC correlate linearly with gene expression. Due to the stronger correlation with SSC, we decided to normalize fluorescence values by dividing, for each cell the fluorescence readout by the SSC readout. (**c**) CV as function of mean expression levels from 5xBS-*CYC180*pr for different inputs. The experiment is the same as shown in **Fig 3a**, without normalization by SSC. The data shows that the qualitative relationships are not affected by normalization.

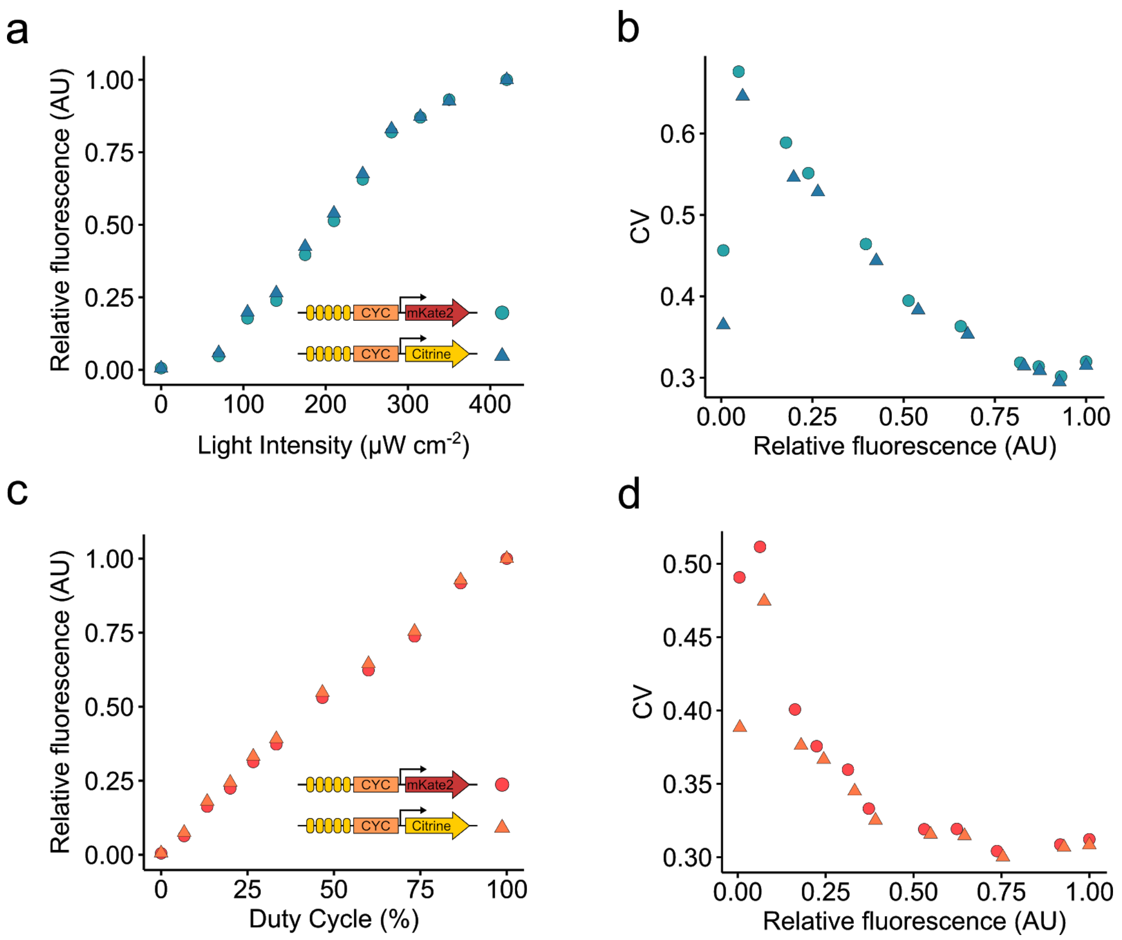

Supplementary Figure 10. Equivalence of mKate2 and mCitrine reporters used for dual-color noise decomposition. AM (**a**,**b**) and PWM (**c**,**d**) experiments were performed with the diploid yeast strain DBY110 expressing both mKate2 and mCitrine from 5xBS-*CYC180*pr. Dose response curves (**a**,**c**) were normalized by dividing each measured value by the maximum value obtained for a given FP in order to reach equivalent fluorescence values for mKate2 and mCitrine. Cells were illuminated under the conditions depicted on the x-axis for 6 h. The light intensity and period for PWM were 420 µW cm^{-2} and 30 min. (**b**,**d**) CV is plotted against mean expression for the same experiment shown in (a) and (c) to analyze the equivalence of FP distributions. The CV differs under non-induced conditions. This likely results from differences in cellular autofluorescence in both fluorescence channels.

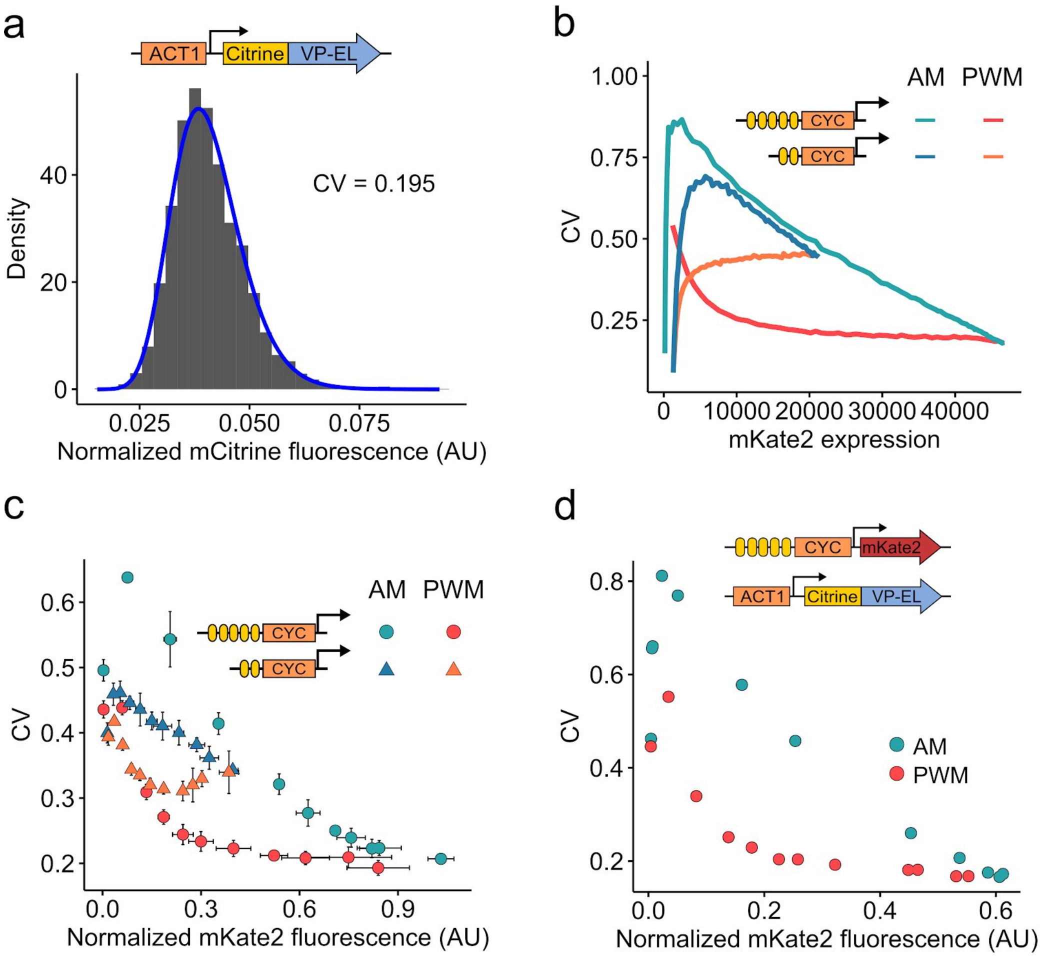

Supplementary Figure 11. Measurement and modeling of VP-EL222 variability. (**a**) Distribution of SSC-normalized mCitrine fluorescence as measured by flow cytometry. Cells express mCitrine-VP-EL222 under control of the *ACT1* promoter. The line represents the fit of a log-normal distribution to the data. The CV of the fitted distribution is shown on the graph. (**b**) The CV-mean expression relationships as calculated from the model for 5xBS-*CYC180*pr and 2xBS-*CYC180*pr are shown. The response to AM (blue) and PWM (red/orange) was evaluated. PWM conditions: 30 min period, 420 μW cm^{-2} light intensity. (**c**) The CV is plotted as a function of mean expression from 5xBS-*CYC180*pr (circle) and 2xBS-*CYC180*pr (triangle) in response to AM (blue) and PWM (red/orange). For 5xBS-*CYC180*pr, results and induction conditions are the same as in **Fig. 3a** with a 30 min PWM period. Results for 2xBS-*CYC180*pr were obtained under the same conditions. Data represents the mean and s.d. of three independent experiments. (**d**) The CV is plotted as a function of mean expression for mCitrine-VP-EL222 mediated expression from 5xBS-*CYC180*pr under AM and PWM. Cells were analyzed after 2h of induction. The light intensity and period for PWM were 350 μW cm^{-2} and 30 min. Pulse durations were: 0, 1, 2, 4, 6, 8, 10, 14, 18, 22, 26, 30 min. Light intensities for AM were: 0, 7, 14, 28, 42, 56, 70, 105,140, 210, 280, and 350 μW cm^{-2}.

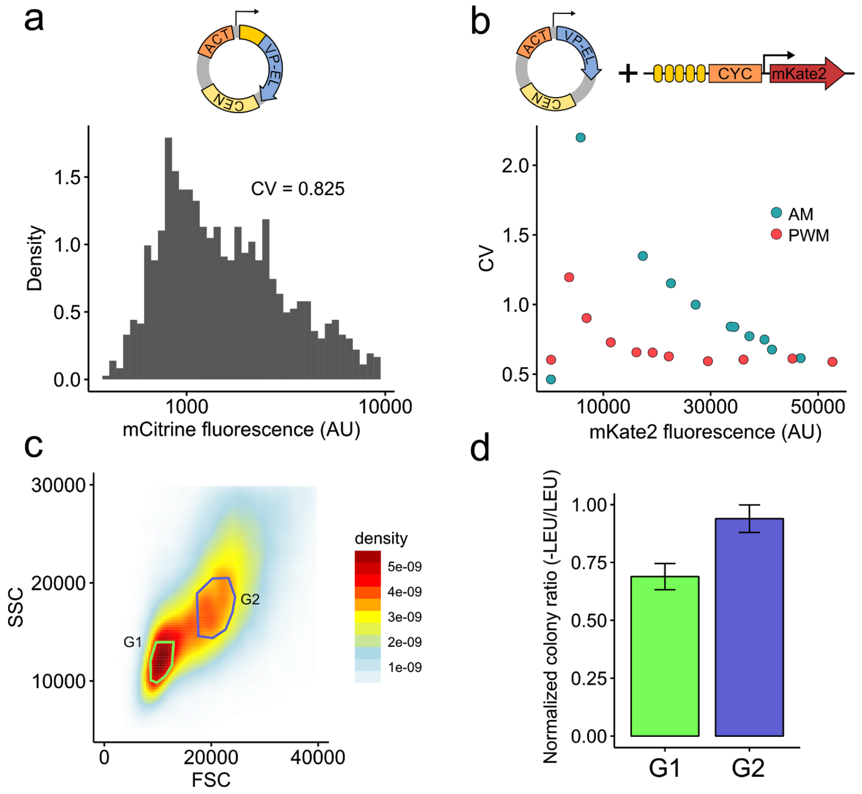

Supplementary Figure 12. Effects of VP-EL222 expression from centromeric plasmid. (**a**) Distribution of mCitrine fluorescence as measured by flow cytometry. Cells express mCitrine-VP-EL222 under control of the *ACT1* promoter from a centromeric plasmid (DBY128). mCitrine fluorescence was not normalized due to a low correlation of fluorescence with SSC. The CV of the fluorescence distribution is shown on the graph. (**b**) The CV is plotted as a function of mean expression from 5xBS-*CYC180*pr in response to AM (blue) and PWM (red) in a strain expressing VP-EL222 from a centromeric plasmid (DBY112). Cells were induced for 6 h before analysis. The following induction conditions were used: AM: 0, 35, 70, 105, 140, 175, 210, 245, 280, 350, 420 µW cm^{-2} light intensity. PWM: 420 µW cm^{-2} light intensity; 45 min period; 0, 3.3, 6.7, 13.3, 20, 26.7, 33.3, 46.7, 60, 90, 100 % duty cycle. (**c**) and (**d**) For strains containing centromeric VP-EL222 plasmids, we found that a subpopulation of yeast cells (G2 in (c)) defined based on forward and side scatter showed a higher percentage of light-responsive cells than the whole population. In order to verify our assumption that the responsiveness results from a higher degree of plasmid retention and justify the use of this population in our experiments, we compared plasmid retention / *LEU2* marker expression in these population. Cells of the two populations shown in (c) were sorted and plated on SD plates with or without L-Leucine (LEU / -LEU). Sorting was performed with strains DBY112 (VP-EL222 on centromeric plasmid) and DBY43 (stable integration of VP-EL222). Colonies were counted after 3 days of growth. Colony ratios between -LEU and LEU plates for DBY112 relative to the values obtained for DBY43 are shown in (d) for the two populations marked in (c). The data indicates relatively low plasmid loss in population G2 and increased plasmid loss in population G1. Data represents mean and s.d. of two technical replicates of yeast dilution and plating for a single sorting experiments.

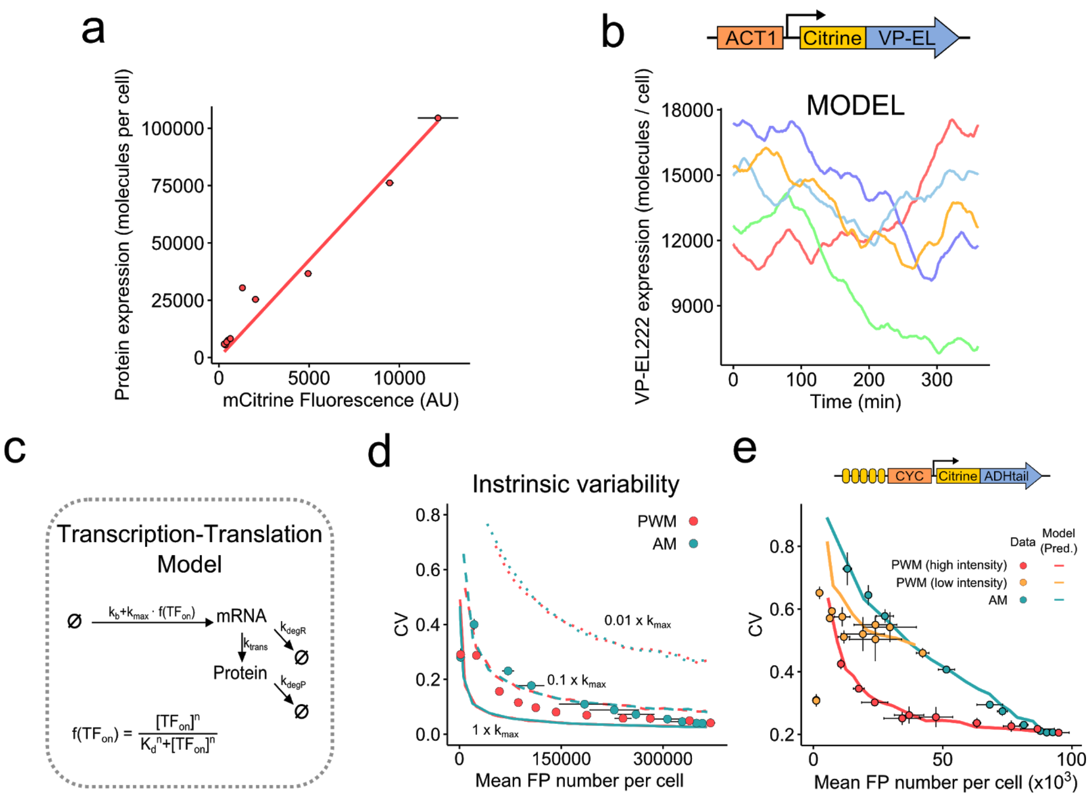

Supplementary Figure 13. Additional data related to the stochastic modeling approach. (**a**) A calibration curve relating the measured fluorescence of reference strains to their know protein copy numbers. See **Supplementary Table 5** for protein identities and copy numbers. For details see **Supplementary Note 4**. Data represents mean and s.d. of two independent experiments, line represents the result of a zero-intercept linear regression. (**b**) Simulated single-cell traces for VP-EL222 expression. See **Supplementary Note 4** for details on modeling. (**c**) Graphical representation of reporter gene expression modeled using a simple transcription-translation model (see **Supplementary Note 4** for details). (**d**) Modeling results using the transcription-translation model (lines) and experimental data (circles) showing intrinsic contributions to gene expression variability for AM (blue) and PWM (red) (see **Supplementary Table 6** for model parameters). A dual color reporter experiment was simulated for 6h and the noise decomposition procedure was applied to the simulated data as described in the Methods section for the experimental data. Simulations were performed for different values of the maximal transcription rate k_{max} (line type, relative change is indicated next to the lines, basal transcription rate and the translation rate were adjusted to result in the same maximal FP expression level). Experimental results are the same as shown in **Fig. 3b**. (**e**) Model predictions for a strain expressing the destabilized Citrine FP under control of the 5xBS-*CYC180*pr (DBY149) using different types of light induction (as indicated on the plot). Period and light intensity for PWM were 30 min and either 140 μW cm^{-2} (yellow) or 420 μW cm^{-2} (blue). Light intensities and duty-cycles were the same as shown in **Fig. 2b,c**. Cellular fluorescence was normalized by side-scatter measurements to reduce the influence of cell size (Methods) and values were multiplied by a constant to bring experimental and modeling results to the same maximal value. Data represents mean and s.d. of two independent experiments. See **Supplementary Table 6** for model parameters.

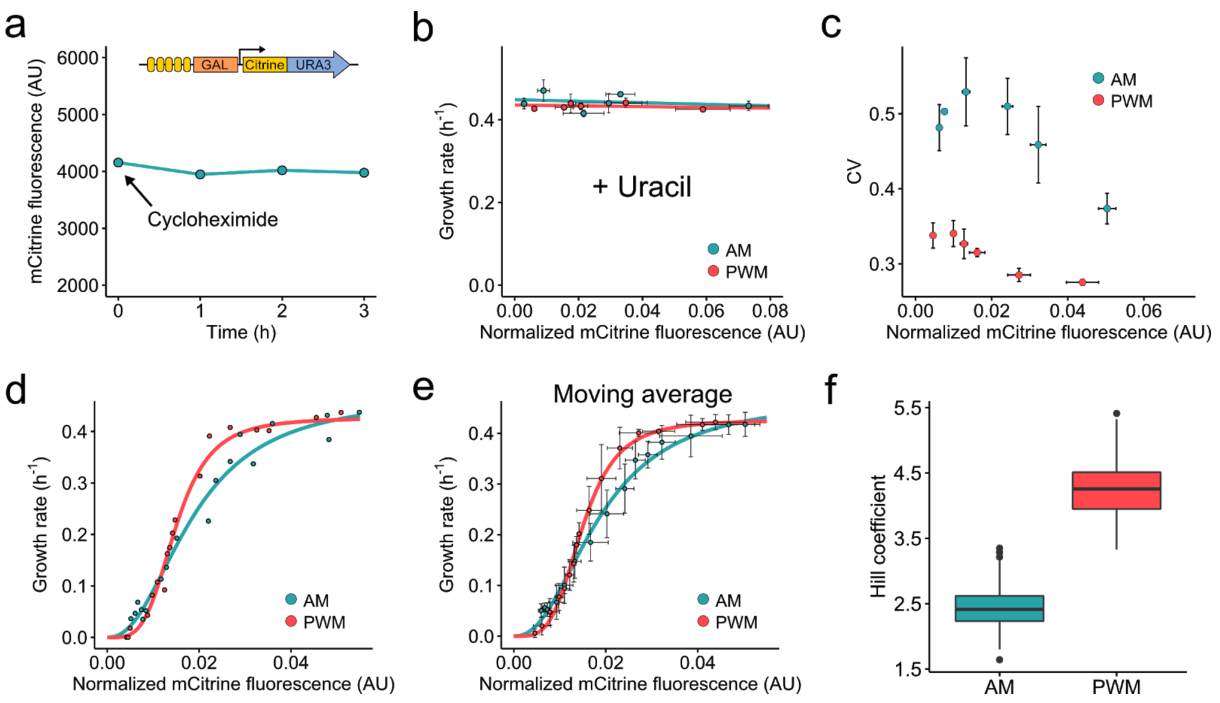

Supplementary Figure 14. Additional data related to measuring the effects of *URA3* expression on growth (Fig. 5). (**a**) Stability of mCitrine-labeled Ura3p. A strain expressing mCitrine-Ura3p from 5xBS-*GAL1*pr was grown under blue light illumination (162 μW cm^{-2}) for 14 h. Cycloheximide was added to the culture media to a final concentration of 0.1 mg/ml and fluorescence was measured over 3h. (**b**) Effect of light-induced URA3 expression on cellular growth in uracil-rich SD medium. The same strain as in (a) was grown for 19 h under multiple blue-light illumination conditions. Cell growth was measured over the last 5 h of the experiment. AM (blue) was performed with light intensities ranging from 0 to 162 μW cm^{-2} and PWM (red) was performed with a 30 min period and a light intensity of 420 μW cm^{-2}. Data represents mean and s.d. of two independent experiments. Lines represent results of linear regressions to the experimental data. (**c**) CV as a function of mean mCitrine expression regulated by AM (blue) and PWM (red) in a strain expressing mCitrine-Ura3p from 5xBS-*GAL1*pr. Data is derived from the experiment shown in Fig. 5a. Data represents the mean and s.e.m. of three independent experiments. (**d**) Data of all individual experiments summarized in Fig. 5a. (**e**) To visualize the differences in dose-response curves shown in (d), the data was smoothed using a moving average with a window size of three data points along the x-axis. Data represents mean and s.d. for each set of three data points. (**f**) In order to evaluate differences in hill coefficient estimates between hill-function fits (for AM and PWM) to the experimental data shown in (d), fitting was performed for 500 bootstrap samples. Distributions of hill coefficient estimates from these fits are presented as box plots. Outliers (dots) are defined as values with a distance from the median greater than 1.5 times the interquartile range.

Supplementary Note 1: Mathematical Modeling of the VP-EL222 mediated expression

In the following section, the specifics about the ordinary differential equation (ODE) model and parameter inference are explained.

A simple ODE model describing VP-EL222 mediated gene expression

In order to obtain a quantitative understanding of the VP-EL222 based gene expression system in *S. cerevisiae*, we constructed a mathematical model of this process. The main purpose of the model is to describe the dynamics of the system in order to understand how dynamic inputs can be used to shape the gene expression output. We thus decided to employ a simplistic gene expression model consisting of three ODEs. These ODEs describe:

(1) Light dependent activation and of the TF VP-EL222.
(2) TF-dependent transcription and mRNA degradation.
(3) Protein translation and degradation.

For simplicity, we assume that TF multimerization and promoter binding occur on fast timescales compared to the transcription process and we thus use Hill-type kinetics to model the effect of activated VP-EL222 (TF_{on}) on the transcription rate.

The model is described by the following ODEs (I denotes the blue light input and TF_{tot} denotes the total amount of cellular VP-EL222):

$$(1)\ \frac{dTF_{on}}{dt} = I \cdot k_{on} \cdot (TF_{tot} - TF_{on}) - k_{off} \cdot TF_{on}$$

$$(2)\ \frac{dmRNA}{dt} = k_{basal} + k_{max} \cdot \frac{TF_{on}^n}{K_d^n + TF_{on}^n} - k_{degR} \cdot mRNA$$

$$(3)\ \frac{dProtein}{dt} = k_{trans} \cdot mRNA - k_{degP} \cdot Protein$$

Fitting model parameters to experimental data

The model possesses 10 parameters (9 rate parameters and the total concentration of the TF / VP-EL222 (TF_{tot})). The value TF_{tot} acts as a scaling factor that can be completely compensated for by changes in other parameters (k_{on} and K_d) and does not affect the dynamics of the system. We thus fixed this value to 2000 molecules/cell. All characterization experiments were performed using the VP-EL222 mediated expression of the stable fluorescent protein (FP) mKate2. We thus equate the protein degradation rate (k_{degP}) to the

cellular growth rate of 0.007 min^{-1} (results of growth rate measurements are shown in **Supplementary Fig. 1**). Thus, we end up with 8 free parameters that need to be estimated.

For this purpose, we performed three characterization experiments using the strain DBY43, expressing mKate2 from the 5xBS-*CYC180* promoter.

1. We performed time-course measurements of mKate2 expression under constant illumination conditions. This experiment was performed to elucidate the kinetics of VP-EL222 activation and (to a larger extent) that of mRNA accumulation / degradation. The kinetics of protein accumulation are given by having fixed k_{degP}.
2. We analyzed the dependence of mKate2 expression on light intensity, i.e. AM. This experiment gives us information about the mapping of light intensity to active transcription factor and finally transcription / protein expression.
3. We analyzed the dependence of mKate2 expression on the duty cycle in a PWM experiment with a short, 7.5 min period. The rationale behind this experiment is that it provides us with information about the kinetics of VP-EL222 activation and deactivation.

The results of these experiments are shown in **Supplementary Fig. 2**. We note again, that we are mainly interested in the dynamics of the gene expression system and not the absolute values of cellular mRNA or protein contents. For the model fitting, we thus assume a direct relation between fluorescence measured by flow cytometry and protein expression in the model.
Parameters were estimated by fitting the model to the mean of three independent experiments of each class of characterization experiments. To do so, we used a simplex-based search (Nelder-Mead algorithm, “fminsearch” function in Matlab) to minimize the sum of squared residuals (SSR) between the model and the data. This procedure was performed for different initial parameter values. The parameters resulting in the minimal SSR between all runs were used in this study and are reported in **Supplementary Table 1**. The model fits are shown in **Supplementary Fig. 2**.

Refitting of promoter-specific model parameters

In order to describe protein expression from the 2xBS-*CYC180* promoter with the mathematical model identified above, we need to re-fit the promoter-specific parameters, k_{basal}, k_{max}, K_d, and n. To do so, we performed two characterization experiment for this promoter, namely we measured the expression response to AM and PWM with a 7.5 min period. Model fitting was performed as described above. Experimental results and fits are shown in **Supplementary Fig. 6**. We found that the fit to the PWM experiment with a 7.5 min period was worse for the 2xBS-*CYC180* than for the 5xBS-*CYC180* promoter (**Supplementary Fig. 6**). In contrast, the response to PWM with a 30 min period was predicted well by the model for both promoters (**Fig. 2b**, **Supplementary Fig. 7**). These results could indicate promoter-dependent differences in the expression response to short (and possibly low amplitude) TF pulses that cannot be recapitulated by the simple gene expression model. Such differences have been previously observed for natural promoters [3] .

Estimating nascent RNA accumulation

Single-molecule FISH (smFISH) allows for the quantification of nascent transcripts, which is a fast readout of VP-EL222's transcriptional activity. We performed an smFISH experiment in which we measure the transcriptional response of 5xBS-*CYC180*pr to a 20 min light pulse (**Supplementary Fig. 3**). To evaluate whether the identified model parameters describing VP-EL222 activity and the transcription process are consistent with this data, we introduce an ODE describing nascent RNA accumulation:

$$(4)\ \frac{dNascent}{dt} = k_{basal} + k_{max} \cdot \frac{TF_{on}^n}{K_d^n + TF_{on}^n} - k_{esc} \cdot Nascent$$

Here, TF-dependent nascent RNA production is modeled using Hill-type kinetics with the same parametrization as for mRNA production (**Equation 2**, **Supplementary Table 4**). The rate at which a nascent RNA escapes from the transcription site (k_{esc}) is given by the RNA dwell-time which includes elongation and termination, leading to k_{esc} = (elongation time)$^{-1}$ + (termination time)$^{-1}$. The termination time was set to the literature value of 70 seconds [4] and the elongation time was set to 100 seconds based on the transcript length of 2000 bases and an average elongation rate of 20 bases per second [4]. We found that the predicted dynamics of nascent RNA accumulation closely resemble the experimental data (**Supplementary Fig. 3c**). We note that this model is very simplistic - it does for example assume that nascent RNAs are observable (via smFISH) directly after transcription initiation.

Supplementary Note 2. Using the ODE model to analyze functional regimes for PWM

The goal of PWM in this study is to regulate TF activity in a pulsatile fashion, while leading to close to constant protein levels over time at steady state. We used the mathematical model to analyze how these properties are affected by different parameters, mainly k_{off}, k_{degP}, and the PWM period. In order to ensure that we are not analyzing transient model behavior, all metrics described below were calculated after running the model for a simulated time of 720 min.

Effects of pulsatile TF regulation via PWM can be expected to be most pronounced when the concentration of active TF directly follows the light input, meaning that cellular TF activity itself shows either the maximal desired value or its basal level at any given time. However, in every realistic scenario, the temporal TF activity will deviate from this behavior to an extent that depends on the kinetics of TF activation / deactivation as well as the PWM period. We hence analyzed how the PWM period and the TF deactivation rate (k_{off}) affect TF pulsing. In order to quantify this behavior, we use a tracking score defined by the ratio between the integrated TF activity during the light pulse and the whole period (**Fig. 1f**). This metric is 1 if the TF activity perfectly tracks the input and equals the duty-cycle if TF activity does not change over the PWM period. We calculated values of this metric for a duty cycle of 50% (**Fig. 1f**). As expected, the model shows that longer PWM periods are required with decreasing k_{off} to achieve a similar tracking score. The model further shows that the inferred rate of k_{off} for VP-EL222 (0.34 min^{-1}, equivalent to a on-state half-life of about 2 min) is sufficiently large for performing PWM with reasonable periods. For a period of 30 min cellular TF activity is predicted to be at its maximal level during much of the light pulse and to return to basal levels in the dark before the next pulse (**Fig. 1f**). In contrast, when the period is reduced to 7.5 min, TF activity is predicted to reach its maximal activity before the end of the light pulse and to not return to the basal level in the dark (**Fig. 1f**). This leads effectively to gene regulation via mixed contributions of constant TF activity and weak pulsing. We found experimentally that this difference has strong functional consequences for the ability to use PWM for gene co-regulation (**Fig. 2d, Supplementary Fig. 8**) and noise reduction (**Fig. 3a,e**).

The model shows that pulsatile TF regulation can be more easily achieved with long PWM periods. However, using long PWM periods to regulate gene expression can potentially result in significant temporal fluctuation on the protein level, which is often not desirable. We thus sought to quantify the temporal response of protein expression to PWM. We use a score defined by the ratio of the maximal expression difference during the period divided by the mean expression level (**Fig. 1g**). Using this score, we found that even for a 30 min period, temporal changes in protein expression at steady state are expected to be minor for a wide range of protein half-lives (**Fig. 1g**). We confirmed experimentally that there is no measurable input tracking for a stable fluorescent protein (**Fig. 1g**). For the median protein half-life of ≈40 min in *S. cerevisiae* [5], PWM is predicted to lead to a maximal temporal fluctuation of about 6% for a 10% duty cycle. We show experimentally that there is indeed relatively low input tracking for a destabilized fluorescent protein with a half-life of ≈30 min (or 23 min if dilution by cell growth is included) (**Fig. 1g, Supplementary Fig. 4a**). We note that the expected fluctuations are also affected by the mRNA degradation rate. Parameter

estimation resulted in a value of 16.5 min for the mRNA half-life, which is close to the median half-life in *S. cerevisiae* (10 - 20 min) [6,7]. Thus, modeling suggests that VP-EL222 should enable PWM-based regulation of a large percentage of yeast proteins. For very short-lived proteins, the system would need to be optimized by introducing mutations that increase the dark-reversion rate. Such mutations were identified previously [8]. We further note that VP-EL222 was previously employed in higher eukaryotes [9], where both mRNA and protein degradation rates were measured to be significantly lower than in *S. cerevisiae* [10,11]. It is thus likely that PWM can be very successfully applied in these organisms and that PWM should also be possible with other tools for gene expression regulation that work on longer time-scales.

Supplementary Note 3: Modeling the effects of VP-EL222 variability

Previous studies suggest that two major sources of extrinsic gene expression variability in *S. cerevisiae* are heterogeneity in TF expression and the cell cycle [12,13]. Due to the fact that we directly affect TF dynamics, we thought to introduce cell-to-cell variability in TF concentration to our model and analyze the resulting CV-mean relationship for AM and PWM. As performed in other studies [14], we modeled protein / TF variability by running 10,000 ODE simulations differing only in the value for TF_{tot} for each input condition (consequences of this modeling choice are described below). Here, each simulation represents a single cell.

To do so, we first measured the fluorescence distribution of mCitrine tagged VP-EL222 to estimate heterogeneity of TF expression. We find that this distribution can be well described by a log-normal distribution with a CV of roughly 0.2 (**Supplementary Fig. 11a**). We then drew values for TF_{tot} from a log-normal distribution with a CV of 0.2 and a mean value of 2000 (the TF_{tot} value used for parameter estimation) and ran ODE simulations for a simulated time of 360 min. We ran simulations for different types of inputs (AM, and PWM with a 7.5, 15, and 30 min period) and different promoters (5xBS-*CYC180*pr and 2xBS-*CYC180*pr). Results of these simulations are shown in **Fig. 3e** and **Supplementary Fig. 11b.**

We found that the model can qualitatively recapitulate the CV-mean relationship for gene expression regulated by both AM and PWM. It also recapitulates the tunability of gene expression variability by changes in PWM period (see also **Fig. 3f**). In addition, the model predicts a reduced noise attenuation by PWM for the 2xBS-*CYC180*pr compared to the 5xBS-*CYC180*pr, which is verified by our experimental results (**Supplementary Fig. 11b,c**). This result shows the importance of working with "promoter-saturating-inputs" for maximal noise reduction by PWM (see **Fig. 3d,f** for an illustration and **Fig. 2b** for input-output functions for both promoters).

However, quantitatively, the model overestimated cell-to-cell variability in gene expression. This is likely a consequence of the model assumption that the TF concentration is fixed in each cell over the 6 h experiment. Indeed, when we compare the simulation results to measurements taken after 2 h of induction (**Supplementary Fig. 11d**), we find a better quantitative agreement. Furthermore, the model does not take into account intrinsic variability which is non-negligible at lower induction levels (**Fig. 3b,c**). We thus expect quantitative and qualitative differences between the model and the data for low expression levels, which can be seen in **Supplementary Fig. 11b,c**. To account for these shortcomings of the ODE modeling approach, we extended the modeling efforts by performing stochastic simulation and found that a simple stochastic model can quantitatively reproduce the observed cell-to-cell variability (see **Supplementary Note 4**).

Supplementary Note 4: Stochastic Modeling of VP-EL222 mediated expression

As stated in **Supplementary Note 3**, the approach to modeling expression variability by changing initial conditions / the concentration VP-EL222 molecules per cell is highly simplistic and does not allow for a quantitative description of the experimental results due to two main shortcomings. First, we assumed that cellular VP-EL222 expression stays constant over the experimental time-scale of 6h, leading to an overestimation of expression variability. Second, while extrinsic variability is dominant for intermediate to high expression levels, we found substantial intrinsic variability as well as PWM mediated variability reduction at low expression levels (**Fig. 3b**). We thus sought to investigate whether a slightly more detailed, stochastic model can quantitatively recapitulate the experimentally observed expression variability. In the following sections, details on the modeling approach are presented.

Calibration of protein copy numbers using a standard curve

In contrast to ODE simulations, the behaviour of stochastic models can be highly dependent on the actual copy number values of molecular species. We thus sought to estimate the average cellular protein copy-numbers of (mCitrine-labeled-)VP-EL222 and the reporter proteins expressed from VP-EL222 activated promoters. Following the approach of Zechner *et al.* [15], we tagged multiple proteins genomically with the mCitrine FP (chosen over mKate2 due to its fast maturation rate) and used the resulting strains to construct a calibration curve that maps arbitrary fluorescent values to protein copy numbers per cell. We selected proteins with measured / estimated copy numbers per cell ranging from 5,000 to 100,000 proteins based on the unified protein abundance dataset presented in ref. 16. Selected proteins and their copy numbers are summarized in **Supplementary Table 5**. To obtain the calibration curve, cells were grown in SD-medium overnight, diluted to an OD_{700} of 0.01 and grown for 6 h before cellular fluorescence was measured using flow cytometry. The fluorescent value obtained for the wild-type BY4741 strain was subtracted from the fluorescence values of the reference strains and a mapping between average protein copy numbers and measured average fluorescence was established by fitting a zero-intercept linear regression model (**Supplementary Fig. 13a**).
The fluorescence of a strain expressing mCitrine-labeled VP-EL222 under the control of the *ACT1* promoter (DBY105) was measured under the same conditions as the reference strains, leading to an estimate of average VP-EL222 copy number of ≈12,300 proteins per cell. We further measured the mCitrine fluorescence of a dual color reporter strain (DBY110, expressing both mCitrine and mKate2 under control of the 5xBS-*CYC180* promoter) after blue light illumination (420 µW cm^{-2}) for 6 h, which resulted in an estimate of average reporter copy number of ≈370,000 proteins per cell.

Modeling of VP-EL222 expression

In order to model dynamic changes in VP-EL222 concentration, we assume that the temporal evolution of VP-EL222 and the resulting cell-to-cell variability results from intrinsic transcriptional dynamics. Given that several constitutively expressed genes in *S. cerevisiae* were shown to be transcribed in uncorrelated single transcription events [4,17], we describe

VP-EL222 expression and activation using the following reaction, where **I** denotes the light input:

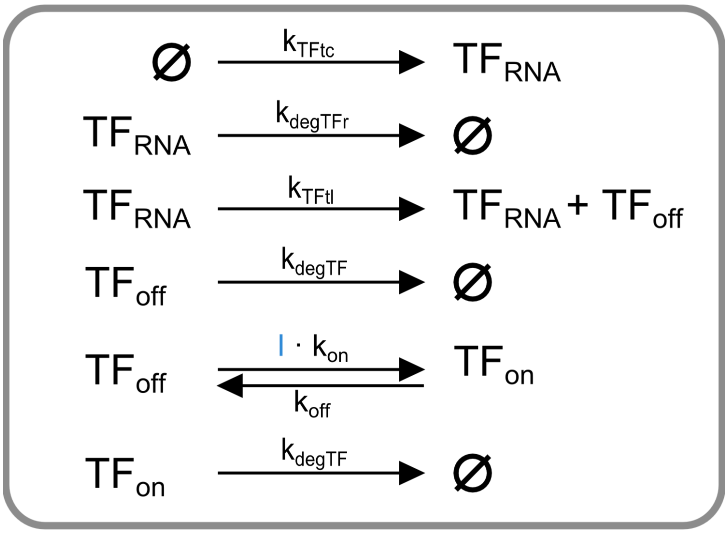

VP-EL222 expression is characterized by four parameter, namely the transcription rate (k_{TFtc}), the translation rate (k_{TFtl}) and mRNA and protein degradation rates (k_{degTFr} and k_{degTF}). We found that the (mCitrine-labeled-)VP-EL222 protein is stable in *S. cerevisiae* and k_{degTF} was thus set to the cellular growth rate of 0.007 min^{-1}. k_{degTFr} was set to 0.063 min^{-1} based on a literature value regarding the half-life of mRNAs with the same terminator sequence [18]. Having these parameters fixed, k_{TFtl} and k_{TFtc} can be calculated based on the VP-EL222 copy number mean (<TF>, see 'Calibration of protein copy numbers using a standard curve') and variance (σ^2_{TF}, see **Supplementary Fig. 11a)** using the following equations [19]:

$$k_{TFtl} = (k_{degTF} + k_{kdegTFr}) \cdot (\frac{\sigma^2_{TF}}{<TF>} - 1)$$

$$k_{TFtc} = \frac{<TF> \cdot k_{degTFr} \cdot k_{degTF}}{(1 - \frac{\sigma^2_{TF}}{<TF>}) \cdot (k_{degTFr} + k_{degTF})}$$

This results in k_{TFtl} = 32.6359 min^{-1} and k_{TFtc} = 0.1660 min^{-1}. In order to model VP-EL222 expression in the diploid dual color reporter strain (DBY110) which expresses VP-EL222 from two identical copies, the rate k_{TFtc} was doubled. **Supplementary Fig. 13b** shows simulated single cell traces showing temporal changes in VP-EL222 protein copy.

Modeling of reporter gene expression

We recently found that VP-EL222 mediated transcription occurs in bursts whose timing as well as duration are affected by VP-EL222 activity [20]. Thus, we modeled reporter gene transcription based on a two-state promoter model [21], with VP-EL222 activity positively regulating promoter activation (switching from a repressed to a transcription competent state) and negatively regulating promoter deactivation. We compared this model to a

standard transcription-translation model in which the transcription rate directly depends on active VP-EL222 concentration (copy number per cell). The models consist of the following reactions:

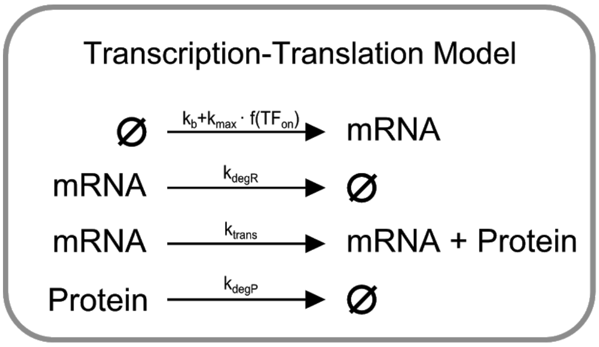

$$f(TF_{on}) = \frac{[TF_{on}]^n}{K_d^n + [TF_{on}]^n}$$

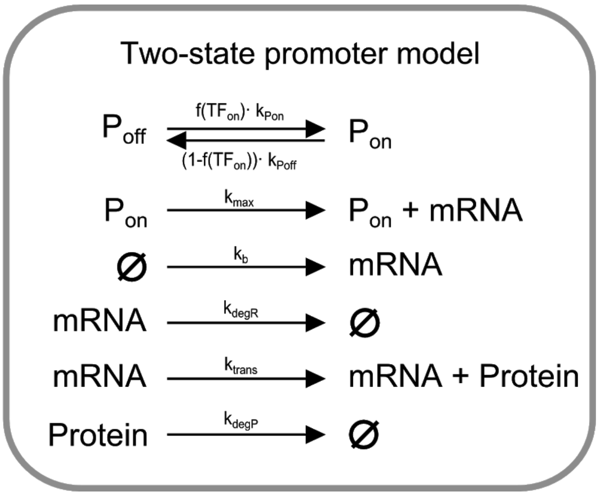

To achieve an input-output behaviour comparable to our experimental data, the dependence of transcription and promoter switching on active VP-EL222 concentration was modeled using a hill equations with parameters derived from the fitted ODE model (**Supplementary Note 1**). K_d was adjusted according to VP-EL222 mean copy numbers in the stochastic model.

In order to model the dual color reporter experiment, we simulate the response of two identical but independent promoters/reporters. In order to decompose intrinsic and extrinsic contributions to gene expression variability, the variability decomposition procedure [22] was applied to the simulated data as described in the Methods section for the experimental data. For all simulation, TF expression (see above) was first simulated for 600 min to reach steady state, before simulating the actual light induction experiments.

In order to bring into agreement the measured reporter protein copy numbers (see above) and the output of the two-state promoter model, we used parameters derived from the ODE model and solely adjusted the translation rate k_{trans}, which resulted in a good fit to the experimental data (**Fig. 4b**). For the the transcription-translation model, we analyzed multiple combinations of transcription rate (k_{basal} and k_{max}) and translation rate (k_{trans}) (**Supplementary Fig. 13 c,d**). All other model parameters are derived from the ODE model fits (**Supplementary Note 1)**. Parameter values used for all simulations are summarized in **Supplementary Table 6**.

In **Figure 4** and **Supplementary Fig. 13** the mean-CV relationship of protein expression resulting from model simulations was compared to cellular fluorescence data that was normalized by side-scatter measurements to reduce the influence of cell size (Methods). Fluorescence values were multiplied by a constant to bring experimental and modeling results to the same maximal value.

Due to high copy numbers for the reporter protein, we used a hybrid model approach in which reporter protein translation and degradation was simulated using an ODE and all other reactions were simulated using the stochastic simulation algorithm (SSA) [23]. We used a custom implementation (provided by Jan Mikelson, ETH Zürich) of the SSA simulation combined with a ODE solver (CVODE package for C++). Since the stochastic part does not depend on the continuous dynamics, the simulation performed the common SSA steps for the stochastic part and reinitialized the ODE solver after each reaction to compute the deterministic dynamics in between the stochastic reactions.

Supplementary Note 5: DNA sequences

Details about the DNA constructs used in this study are described below. The color coding of the sequences corresponds to colors used in the preceding text.

VP-EL222 constructs

pDB58; pDB116 / *ACT1*pr - VP-EL222 - *CYC1*term

pDB58 was used to construct yeast strains expressing VP-EL222 from the *LEU2* locus. It consists of the ***ACT1* promoter**, the coding sequence for **NLS**-**VP16**-**EL222** (derived from pVP-EL222 [9]) and the ***CYC1* terminator** integrated into an integrative vector based on the pRS vector series. The same construct was integrated into the centromeric plasmid pRG215 [2] to generate pDB116.

tctagagaagcgggtaagctgccacagcaattaatgcacaacatttaacctacattcttccttatcggatcgtcaaaacc
cttaaaaagatatgcctcaccctaacatattttccaattaaccctcaatatttctctgtcacccggcctctattttccattttctt
ctttacccgccacgcgttttttctttcaaatttttttcttccttcttttttcttccacgtcctcttgcataaataaataaaccgttt
tgaaaccaaactcgcctctctctctcctttttgaaatatttttgggtttgtttgatcctttccttcccaatctctcttgtttaatatat
attcatttatatcacgctctctttttatcttcctttttttcctctctcttgtattcttccttcccctttctactcaaaccaagaagaaa
aagaaaaggtcaatctttgttaaagaataggatcttctactacatcagcttttagatttttcacgcttactgctttttcttccca
agatcgaaaatttactgaattaacaactagtATGGGCCCTAAAAAGAAGCGTAAAGTCGCCCCCCC
GACCGATGTCAGCCTGGGGGACGAGCTCCACTTAGACGGCGAGGACGTGGCGATGG
CGCATGCCGACGCGCTAGACGATTTCGATCTGGACATGTTGGGGGACGGGGATTCCC
CGGGTCCGGGATTTACCCCCCACGACTCCGCCCCCTACGGCGCTCTGGATATGGCCG
ACTTCGAGTTTGAGCAGATGTTTACCGATGCCCTTGGAATTGACGAGTACGGTGGGG
AATTCGGGGCAGACGACACACGCGTTGAGGTGCAACCGCCGGCGCAGTGGGTCCTC
GACCTGATCGAGGCCAGCCCGATCGCATCGGTCGTGTCCGATCCGCGTCTCGCCGAC
AATCCGCTGATCGCCATCAACCAGGCCTTCACCGACCTGACCGGCTATTCCGAAGAA
GAATGCGTCGGCCGCAATTGCCGATTCCTGGCAGGTTCCGGCACCGAGCCGTGGCTG
ACCGACAAGATCCGCCAAGGCGTGCGCGAGCACAAGCCGGTGCTGGTCGAGATCCT
GAACTACAAGAAGGACGGCACGCCGTTCCGCAATGCCGTGCTCGTTGCACCGATCTA
CGATGACGACGACGAGCTTCTCTATTTCCTCGGCAGCCAGGTCGAAGTCGACGACGA
CCAGCCCAACATGGGCATGGCGCGCCGCGAACGCGCCGCGGAAATGCTCAAGACGC
TGTCGCCGCGCCAGCTCGAGGTTACGACGCTGGTGGCATCGGGCTTGCGCAACAAG
GAAGTGGCGGCCCGGCTCGGCCTGTCGGAGAAAACCGTCAAGATGCACCGCGGGCT
GGTGATGGAAAAGCTCAACCTGAAGACCAGTGCCGATCTGGTGCGCATTGCCGTCGA
AGCCGGAATCTAActcgagacaggccccttttcctttgtctatatcatgtaattagttatgtcacgcttacattcacg
ccctcctcccacatccgctctaaccgaaaaggaaggagttagacaacctgaagtctaggtccctatttatttttttttaatag
ttatgttagtattaagaacgttatttatatttcaaatttttcttttttttctgtacaaacgcgtgtacgcatgtaacattatactgaa
aaccttgcttgagaaggttttgggacgctcgaaggctttaatttgcaaggttcgcagtttacactctcatcgtcgctctcat
catcgcttccgttgttgttttccttagtagcgtctgcttggtacc

pDB113; pDB131 / mCitrine-VP-EL222

In order to quantify VP-EL222 expression in single cells, a coding sequence for **mCitrine** [24] was inserted upstream of VP-EL222. All other aspects of the sequence are as described above for pDB58 / pDB116

actagtATGGTAAGTAAGGGTGAAGAATTATTCACTGGTGTTGTCCCAATTTTGGTTGAA
TTAGATGGTGATGTTAATGGTCACAAATTTTCTGTCTCCGGTGAAGGTGAAGGTGATG
CTACTTACGGTAAATTGACCTTAAAATTTATTTGTACTACTGGTAAATTGCCAGTTCCA
TGGCCAACCTTAGTCACTACTTTAGGTTATGGTTTGATGTGTTTTGCTAGATACCCAGA
TCATATGAAACAACATGACTTTTTCAAGTCTGCCATGCCAGAAGGTTATGTTCAAGAA
AGAACTATTTTTTTCAAAGATGACGGTAACTACAAGACCAGAGCTGAAGTCAAGTTTG
AAGGTGATACCTTAGTTAATAGAATCGAATTAAAAGGTATTGATTTTAAAGAAGATGG
TAACATTTTAGGTCACAAATTGGAATACAACTATAACTCTCACAATGTTTACATCATGG
CTGACAAACAAAAGAATGGTATCAAAGTTAACTTCAAAATTAGACACAACATTGAAGA
TGGTTCTGTTCAATTAGCTGACCATTATCAACAAAATACTCCAATTGGTGATGGTCCA
GTCTTGTTACCAGACAACCATTACTTATCCTATCAATCTAAGTTATCCAAAGATCCAAA
CGAAAAGAGAGACCACATGGTCTTGTTAGAATTTGTTACTGCTGCTGGTATTACCCAT
GGAATGGACGAATTGTACAAAGGCCCTAAAAAGAAGCGTAAAGTCGCCCCCCCGAC
CGATGTCAGCCTGGGGGACGAGCTCCACTTAGACGGCGAGGACGTGGCGATGGCGC
ATGCCGACGCGCTAGACGATTTCGATCTGGACATGTTGGGGGACGGGGATTCCCCGG
GTCCGGGATTTACCCCCCACGACTCCGCCCCCTACGGCGCTCTGGATATGGCCGACT
TCGAGTTTGAGCAGATGTTTACCGATGCCCTTGGAATTGACGAGTACGGTGGGGAATT
CGGGGCAGACGACACACGCGTTGAGGTGCAACCGCCGGCGCAGTGGGTCCTCGACC
TGATCGAGGCCAGCCCGATCGCATCGGTCGTGTCCGATCCGCGTCTCGCCGACAATC
CGCTGATCGCCATCAACCAGGCCTTCACCGACCTGACCGGCTATTCCGAAGAAGAAT
GCGTCGGCCGCAATTGCCGATTCCTGGCAGGTTCCGGCACCGAGCCGTGGCTGACC
GACAAGATCCGCCAAGGCGTGCGCGAGCACAAGCCGGTGCTGGTCGAGATCCTGAA
CTACAAGAAGGACGGCACGCCGTTCCGCAATGCCGTGCTCGTTGCACCGATCTACGA
TGACGACGACGAGCTTCTCTATTTCCTCGGCAGCCAGGTCGAAGTCGACGACGACCA
GCCCAACATGGGCATGGCGCGCCGCGAACGCGCCGCGGAAATGCTCAAGACGCTGT
CGCCGCGCCAGCTCGAGGTTACGACGCTGGTGGCATCGGGCTTGCGCAACAAGGAA
GTGGCGGCCCGGCTCGGCCTGTCGGAGAAAACCGTCAAGATGCACCGCGGGCTGGT
GATGGAAAAGCTCAACCTGAAGACCAGTGCCGATCTGGTGCGCATTGCCGTCGAAGC
CGGAATCTAActcgag

VP-EL222 - dependent promoter sequences / reporter constructs

For all following promoter sequences, EL222 binding sites (BS; called C120 in the original publication) are underlined and promoter backbones are green. A sequence containing 5 binding sites for EL222 was amplified from pcDNA-C120-mCherry [9]. All other binding site combinations were constructed by oligonucleotide annealing to obtain a single plasmid containing a single EL222 binding site, followed by duplications of this sequence using restriction enzyme cloning.

pDB60 / 5xBS-*CYC180*pr-Kozak-mKate2-*ADH1*t

pDB60 is used to express **mKate2** [25] under control of the 5xBS-*CYC180* promoter. The promoter consists of a sequence containing 5 EL222 binding sites as well as a 180 bp sequence derived from the *CYC1* promoter (***CYC180***). *CYC180* was amplified from BY4741 genomic DNA [26]. A consensus **Kozak** sequence was inserted upstream of the start codon to enhance translation. The mKate2 reporter gene is inserted into pFA6a-*His3MX6* [27] using PacI and AscI sites (upstream of the ***ADH1* terminator**). 5xBS-*CYC180*pr is inserted using HindIII and PacI.
All other VP-EL222 dependent promoters (see below) were characterized using the same plasmid backbone and were integrated into HindIII/PacI digested plasmid.

**aagcttGGGAGATCTTCGCTAGCCTCGAGTAGGTAGCCTTTAGTCCATGCGTTATAGGTA
GCCTTTAGTCCATGCGTTATAGGTAGCCTTTAGTCCATGCGTTATAGGTAGCCTTTAGT
CCATGCGTTATAGGTAGCCTTTAGTCCATGAAGCTTAGACACTAGAGGGACTAGAGT
GCTGACACTACAGGCATATATATATGTGTGCGACGACACATGATCATATGGCATGCAT
GTGCTCTGTATGTATATAAAACTCTTGTTTTCTTCTTTTCTCTAAATATTCTTTCCTTATA
CATTAGGACCTTTGCAGCATAAATTACTATACTTCTATAGACACACAAACACAAATAC
AttaattaaAACAAAATGAGATCTGTTTCTGAATTGATTAAAGAAAACATGCATATGAAGT
TGTATATGGAAGGTACTGTTAACAATCATCATTTCAAGTGTACATCTGAAGGTGAAGG
TAAACCATATGAAGGTACTCAAACTATGAGAATTAAAGCTGTTGAAGGTGGTCCATTG
CCATTTGCTTTTGATATTTTGGCTACTTCTTTCATGTATGGTTCTAAGACTTTCATTAAC
CATACTCAAGGTATTCCAGATTTTTTCAAGCAATCTTTTCCAGAAGGTTTTACTTGGGA
AAGAGTTACTACTTACGAAGATGGTGGTGTTTTGACTGCTACTCAAGATACTTCTTTGC
AAGATGGTTGTTTGATTTACAACGTTAAGATTAGAGGTGTTAATTTTCCATCTAATGGT
CCAGTTATGCAAAAAAAGACTTTGGGTTGGGAAGCATCTACTGAAACTTTGTATCCAG
CTGATGGTGGTTTGGAAGGTAGAGCTGATATGGCTTTGAAATTGGTTGGTGGTGGTCA
TTTGATTTGTAACTTGAAAACTACTTACAGATCAAAAAAGCCAGCTAAGAATTTGAAA
ATGCCAGGTGTTTATTACGTTGATAGAAGATTGGAAAGAATTAAAGAAGCTGATAAAG
AAACTTACGTTGAACAACATGAAGTTGCTGTTGCAAGATATTGTGATTTGCCATCTAA
ATTGGGTCATAGAGGATCCTAGggcgcgccacttctaaataagcgaatttcttatgatttatgatttttattatta
aataagttataaaaaaaataagtgtatacaaattttaaagtgactcttaggttttaaaacgaaaattcttattcttgagtaact
ctttcctgtaggtcaggttgctttctcaggtatagtatgaggtcgctcttattgaccacacctctaccggca**

pDB72 / 5xBS-*GAL1*pr

The design of the VP-EL222 dependent *GAL1*-based promoter is adapted from another synthetic gene expression system presented in ref. 28. The promoter was constructed by exchanging the UAS-GAL region (containing Gal4p binding sites) of the *GAL1* promoter with the 5xBS sequence from pcDNA-C120-mCherry [9].

**aagcttTTTAATTATATCAGTTATTACCCGGTACCCCCCTCGAGGAATTTTCAAAAATTCT
TACTTTTTTTTTGGATGGACGCAAAGAAGTTTAATAATCATATTACATGGCATTACCAC
CATATACATATCCATATACATATCCATATCTAATCTTACTTATATGTTGTGGAAATGTAA
AGAGCCCCATTATCTTAGCCTAAAAAAACCTTCTCTTTGGAACTTTCAGTAATACGCTT
AACTGCTCATTGCTATATTGAAGTGCGGCCGCGGGAGATCTTCGCTAGCCTCGAGTA
GGTAGCCTTTAGTCCATGCGTTATAGGTAGCCTTTAGTCCATGCGTTATAGGTAGCCT
TTAGTCCATGCGTTATAGGTAGCCTTTAGTCCATGCGTTATAGGTAGCCTTTAGTCCAT
GAAGCTTAGACACTAGAGGGACTAGACCGTGCGTCCTCGTCTTCACCGGTCGCGTTC
CTGAAACGCAGATGTGCCTCGCGCCGCACTGCTCCGAACAATAAAGATTCTACAATA
CTAGCTTTTATGGTTATGAAGAGGAAAAATTGGCAGTAACCTGGCCCCACAAACCTTC
AAATGAACGAATCAAATTAACAACCATAGGATGATAATGCGATTAGTTTTTTAGCCTT
ATTTCTGGGGTAATTAATCAGCGAAGCGATGATTTTTGATCTATTAACAGATATATAAA
TGCAAAAACTGCATAACCACTTTAACTAATACTTTCAACATTTTCGGTTTGTATTACTTC
TTATTCAAATGTAATAAAAGTATCAACAAAAAATTGTTAATATACCTCTATACTTTAAC
GTCAAGGAGAAAAAACTATAttaattaa**

pDB107 / 5xBS-*SPO*13pr

In order to achieve light-dependant gene expression with very low basal expression, we inserted EL222 binding sites upstream of the basal *SPO13* promoter [29]. The *SPO13* promoter sequence was amplified from BY4741 genomic DNA.

**aagcttGGGAGATCTTCGCTAGCCTCGAGTAGGTAGCCTTTAGTCCATGCGTTATAGGTA
GCCTTTAGTCCATGCGTTATAGGTAGCCTTTAGTCCATGCGTTATAGGTAGCCTTTAGT
CCATGCGTTATAGGTAGCCTTTAGTCCATGAAGCTTAGACACTAGAGGGACTAGATTT
AGTATCCGTTTAGCTAGTTAGTACCTTTGCACGGAAATGTATTAATTAGGAGTATATTG
AGAAATAGCCGCCGACAAAAAGGAAGTCTCATAAAAGTGTCTAACAGACAATTAGCG
CAATAAGAAGAAAGAAAACGGATTGAAGTTGAGTCGAGAATAATTttaattaa**

pDB67 / 1xBS-*CYC180*pr

**aagcttGCGGCCGCTTCTAGATAGGTAGCCTTTAGTCCATGACTAGAGTGCTGACACTA
CAGGCATATATATATGTGTGCGACGACACATGATCATATGGCATGCATGTGCTCTGTA
TGTATATAAAACTCTTGTTTTCTTCTTTTCTCTAAATATTCTTTCCTTATACATTAGGACC
TTTGCAGCATAAATTACTATACTTCTATAGACACACAAACACAAATACAttaattaa**

pDB99 / 2xBS-*CYC180*pr

aagcttAGCTTGCGTTCGCTACTAGTAGCTAGCCTTTAGTCCATGTCTAGTAGCTAGCCT
TTAGTCCATGTCTAGAGTGCTGACACTACAGGCATATATATATGTGTGCGACGACACA
TGATCATATGGCATGCATGTGCTCTGTATGTATATAAAACTCTTGTTTTCTTCTTTTCTC
TAAATATTCTTTCCTTATACATTAGGACCTTTGCAGCATAAATTACTATACTTCTATAGA
CACACAAACACAAATACAttaattaa

pDB100 / 3xBS-*CYC180*pr

aagcttAGCTTGCGTTCGCTACTAGTAGCTAGCCTTTAGTCCATGTCTAGTAGCTAGCCT
TTAGTCCATGTCTAGTAGCTAGCCTTTAGTCCATGTCTAGAGTGCTGACACTACAGGC
ATATATATATGTGTGCGACGACACATGATCATATGGCATGCATGTGCTCTGTATGTAT
ATAAAACTCTTGTTTTCTTCTTTTCTCTAAATATTCTTTCCTTATACATTAGGACCTTTGC
AGCATAAATTACTATACTTCTATAGACACACAAACACAAATACAttaattaa

pDB101 / 4xBS-*CYC180*pr

aagcttAGCTTGCGTTCGCTACTAGTAGCTAGCCTTTAGTCCATGTCTAGTAGCTAGCCT
TTAGTCCATGTCTAGTAGCTAGCCTTTAGTCCATGTCTAGTAGCTAGCCTTTAGTCCAT
GTCTAGAGTGCTGACACTACAGGCATATATATATGTGTGCGACGACACATGATCATAT
GGCATGCATGTGCTCTGTATGTATATAAAACTCTTGTTTTCTTCTTTTCTCTAAATATTC
TTTCCTTATACATTAGGACCTTTGCAGCATAAATTACTATACTTCTATAGACACACAAA
CACAAATACAttaattaa

pDB102 / 6xBS-*CYC180*pr

aagcttAGCTTGCGTTCGCTACTAGTAGCTAGCCTTTAGTCCATGTCTAGTAGCTAGCCT
TTAGTCCATGTCTAGTAGCTAGCCTTTAGTCCATGTCTAGTAGCTAGCCTTTAGTCCAT
GTCTAGTAGCTAGCCTTTAGTCCATGTCTAGTAGCTAGCCTTTAGTCCATGTCTAGAGT
GCTGACACTACAGGCATATATATATGTGTGCGACGACACATGATCATATGGCATGCAT
GTGCTCTGTATGTATATAAAACTCTTGTTTTCTTCTTTTCTCTAAATATTCTTTCCTTATA
CATTAGGACCTTTGCAGCATAAATTACTATACTTCTATAGACACACAAACACAAATAC
Attaattaa

pDB103 / *TDH3*pr

aagcttTCAGTTCGAGTTTATCATTATCAATACTGCCATTTCAAAGAATACGTAAATAATT
AATAGTAGTGATTTTCCTAACTTTATTTAGTCAAAAAATTAGCCTTTTAATTCTGCTGTA
ACCCGTACATGCCCAAAATAGGGGGCGGGTTACACAGAATATATAACATCGTAGGTG
TCTGGGTGAACAGTTTATTCCTGGCATCCACTAAATATAATGGAGCCCGCTTTTTAAG

**CTGGCATCCAGAAAAAAAAAAGAATCCCAGCACCAAAATATTGTTTTCTTCACCAACCA
TCAGTTCATAGGTCCATTCTCTTAGCGCAACTACAGAGAACAGGGGCACAAACAGGC
AAAAAACGGGCACAACCTCAATGGAGTGATGCAACCTGCCTGGAGTAAATGATGACA
CAAGGCAATTGACCCACGCATGTATCTATCTCATTTTCTTACACCTTCTATTACCTTCT
GCTCTCTCTGATTTGGAAAAAGCTGAAAAAAAAAGGTTGAAACCAGTTCCCTGAAATTA
TTCCCCTACTTGACTAATAAGTATATAAAGACGGTAGGTATTGATTGTAATTCTGTAAA
TCTATTTCTTAAACTTCTTAAATTCTACTTTTATAGTTAGTCTTTTTTTTAGTTTTAAAAC
ACCAAGAACTTAGTTTCGAATAAACACACATAAACAAACAAAAttaattaa**

Further sequences expressed from VP-EL222 dependent promoters

As shown above for mKate2 in pDB60, all sequences were integrated into PacI, AscI digested pFA6a-*His3MX6*-derived plasmids. Furthermore, all sequences possess a Kozak consensus sequence directly upstream of the start codon.

pDB78 / mKate2 - 24xPP7SL

A sequence containing 24 tandem repeats of the **PP7 stem loop** was amplified from pDZ416 [30] and was inserted after the mKate2 stop codon.

**ttaattaaAACAAAATGAGATCTGTTTCTGAATTGATTAAAGAAAACATGCATATGAAGTT
GTATATGGAAGGTACTGTTAACAATCATCATTTCAAGTGTACATCTGAAGGTGAAGGT
AAACCATATGAAGGTACTCAAACTATGAGAATTAAAGCTGTTGAAGGTGGTCCATTGC
CATTTGCTTTTGATATTTTGGCTACTTCTTTCATGTATGGTTCTAAGACTTTCATTAACC
ATACTCAAGGTATTCCAGATTTTTTCAAGCAATCTTTTCCAGAAGGTTTTACTTGGGAA
AGAGTTACTACTTACGAAGATGGTGGTGTTTTGACTGCTACTCAAGATACTTCTTTGCA
AGATGGTTGTTTGATTTACAACGTTAAGATTAGAGGTGTTAATTTTCCATCTAATGGTC
CAGTTATGCAAAAAAAGACTTTGGGTTGGGAAGCATCTACTGAAACTTTGTATCCAGC
TGATGGTGGTTTGGAAGGTAGAGCTGATATGGCTTTGAAATTGGTTGGTGGTGGTCAT
TTGATTTGTAACTTGAAAACTACTTACAGATCAAAAAAGCCAGCTAAGAATTTGAAAA
TGCCAGGTGTTTATTACGTTGATAGAAGATTGGAAAGAATTAAAGAAGCTGATAAAGA
AACTTACGTTGAACAACATGAAGTTGCTGTTGCAAGATATTGTGATTTGCCATCTAAAT
TGGGTCATAGAGGATCCtaaggtacctaattgcctagaaaggagcagacgatatggcgtcgctccctgcag
gtcgactctagaaaccagcagagcatatgggctcgctggctgcagtattcccgggttcattagatcctaaggtacctaa
ttgcctagaaaggagcagacgatatggcgtcgctccctgcaggtcgactctagaaaccagcagagcatatgggctcg
ctggctgcagtattcccgggttcattagatcctaaggtacctaattgcctagaaaggagcagacgatatggcgtcgctc
cctgcaggtcgactctagaaaccagcagagcatatgggctcgctggctgcagtattcccgggttcattagatcctaag
gtacctaattgcctagaaaggagcagacgatatggcgtcgctccctgcaggtcgactctagaaaccagcagagcata
tgggctcgctggctgcagtattcccgggttcattagatcctaaggtacctaattgcctagaaaggagcagacgatatgg
cgtcgctccctgcaggtcgactctagaaaccagcagagcatatgggctcgctggctgcagtattcccgggttcattag
atcctaaggtacctaattgcctagaaaggagcagacgatatggcgtcgctccctgcaggtcgactctagaaaccagca
gagcatatgggctcgctggctgcagtattcccgggttcattagatcctaaggtacctaattgcctagaaaggagcagac
gatatggcgtcgctccctgcaggtcgactctagaaaccagcagagcatatgggctcgctggctgcagtattcccgggt
tcattagatcctaaggtacctaattgcctagaaaggagcagacgatatggcgtcgctccctgcaggtcgactctagaaa**

**ccagcagagcatatgggctcgctggctgcagtattcccgggttcattagatcctaaggtacctaattgcctagaaagga
gcagacgatatggcgtcgctccctgcaggtcgactctagaaaccagcagagcatatgggctcgctggctgcagtattc
ccgggttcattagatcctaaggtacctaattgcctagaaaggagcagacgatatggcgtcgctccctgcaggtcgactc
tagaaaccagcagagcatatgggctcgctggctgcagtattcccgggttcattagatcctaaggtacctaattgcctag
aaaggagcagacgatatggcgtcgctccctgcaggtcgactctagaaaccagcagagcatatgggctcgctggctg
cagtattcccgggttcattagatcctaaggtacctaattgcctagaaaggagcagacgatatggcgtcgctccctgcag
gtcgactctagaaaccagcagagcatatgggctcgctggctgcagtattcccgggttcattagatcccccgggctggc
gcgcc**

pDB110 / mCitrine

**ttaattaaAACAAAATGAAAGGTGAAGAATTATTCACTGGTGTTGTCCCAATTTTGGTTGA
ATTAGATGGTGATGTTAATGGTCACAAATTTTCTGTCTCCGGTGAAGGTGAAGGTGAT
GCTACTTACGGTAAATTGACCTTAAAATTTATTTGTACTACTGGTAAATTGCCAGTTCC
ATGGCCAACCTTAGTCACTACTTTAGGTTATGGTTTGATGTGTTTTGCTAGATACCCAG
ATCATATGAAACAACATGACTTTTTCAAGTCTGCCATGCCAGAAGGTTATGTTCAAGA
AAGAACTATTTTTTTCAAAGATGACGGTAACTACAAGACCAGAGCTGAAGTCAAGTTT
GAAGGTGATACCTTAGTTAATAGAATCGAATTAAAAGGTATTGATTTTAAAGAAGATG
GTAACATTTTAGGTCACAAATTGGAATACAACTATAACTCTCACAATGTTTACATCATG
GCTGACAAACAAAAGAATGGTATCAAAGTTAACTTCAAAATTAGACACAACATTGAAG
ATGGTTCTGTTCAATTAGCTGACCATTATCAACAAAATACTCCAATTGGTGATGGTCCA
GTCTTGTTACCAGACAACCATTACTTATCCTATCAATCTAAGTTATCCAAAGATCCAAA
CGAAAAGAGAGACCACATGGTCTTGTTAGAATTTGTTACTGCTGCTGGTATTACCCAT
GGTATGGATGAATTGTACAAAggcgcgcc**

pDB150 / Destabilized Citrine (Citrine-*ADH1*tail)

The *ADH1* tail sequence [1,2] used for destabilization is highlighted in blue.

**ttaattaaAACAAAATGAGATCTAAAGGTGAAGAATTATTCACTGGTGTTGTCCCAATTTT
GGTTGAATTAGATGGTGATGTTAATGGTCACAAATTTTCTGTCTCCGGTGAAGGTGAA
GGTGATGCTACTTACGGTAAATTGACCTTAAAATTTATTTGTACTACTGGTAAATTGCC
AGTTCCATGGCCAACCTTAGTCACTACTTTAGGTTATGGTTTGATGTGTTTTGCTAGAT
ACCCAGATCATATGAAACAACATGACTTTTTCAAGTCTGCCATGCCAGAAGGTTATGT
TCAAGAAAGAACTATTTTTTTCAAAGATGACGGTAACTACAAGACCAGAGCTGAAGTC
AAGTTTGAAGGTGATACCTTAGTTAATAGAATCGAATTAAAAGGTATTGATTTTAAAGA
AGATGGTAACATTTTAGGTCACAAATTGGAATACAACTATAACTCTCACAATGTTTACA
TCATGGCTGACAAACAAAAGAATGGTATCAAAGTTAACTTCAAAATTAGACACAACAT
TGAAGATGGTTCTGTTCAATTAGCTGACCATTATCAACAAAATACTCCAATTGGTGAT
GGTCCAGTCTTGTTACCAGACAACCATTACTTATCCTATCAATCTAAGTTATCCAAAGA
TCCAAACGAAAAGAGAGACCACATGGTCTTGTTAGAATTTGTTACTGCTGCTGGTATT
ACCCATGGTATGGATGAATTGTACAACACTTGGTCTACTGTTAGAAGATCCGGTTTGG
AAACTCAATTCAGAGCTTTGGACTTCTTCGCCAGAGGTTTGGTCAAGTCTCCAATCAA
GGTTGTCGGCTTGTCTACCTTGCCAGAAATTTACGAAAAGATGGAAAAGGGTCAAATC
GTTGGTAGATACGTTGTTGACACTTCTAAATAAggcgcgcc**

pDB152 / mCitrine-*URA3*

The ***URA3* coding sequence** was amplified from BY4741 genomic DNA. *URA3* and mCitrine are separated by a **linker sequence**.

**ttaattaaAACAAAATGAAAGGTGAAGAATTATTCACTGGTGTTGTCCCAATTTTGGTTGA
ATTAGATGGTGATGTTAATGGTCACAAATTTTCTGTCTCCGGTGAAGGTGAAGGTGAT
GCTACTTACGGTAAATTGACCTTAAAATTTATTTGTACTACTGGTAAATTGCCAGTTCC
ATGGCCAACCTTAGTCACTACTTTAGGTTATGGTTTGATGTGTTTTGCTAGATACCCAG
ATCATATGAAACAACATGACTTTTTCAAGTCTGCCATGCCAGAAGGTTATGTTCAAGA
AAGAACTATTTTTTTCAAAGATGACGGTAACTACAAGACCAGAGCTGAAGTCAAGTTT
GAAGGTGATACCTTAGTTAATAGAATCGAATTAAAAGGTATTGATTTTAAAGAAGATG
GTAACATTTTAGGTCACAAATTGGAATACAACTATAACTCTCACAATGTTTACATCATG
GCTGACAAACAAAAGAATGGTATCAAAGTTAACTTCAAAATTAGACACAACATTGAAG
ATGGTTCTGTTCAATTAGCTGACCATTATCAACAAAATACTCCAATTGGTGATGGTCCA
GTCTTGTTACCAGACAACCATTACTTATCCTATCAATCTAAGTTATCCAAAGATCCAAA
CGAAAAGAGAGACCACATGGTCTTGTTAGAATTTGTTACTGCTGCTGGTATTACCCAT
GGTATGGATGAATTGTACAAAggtgacggtgctggtttaataaacATGTCGAAAGCTACATATA
AGGAACGTGCTGCTACTCATCCTAGTCCTGTTGCTGCCAAGCTATTTAATATCATGCA
CGAAAAGCAAACAAACTTGTGTGCTTCATTGGATGTTCGTACCACCAAGGAATTACTG
GAGTTAGTTGAAGCATTAGGTCCCAAAATTTGTTTACTAAAAACACATGTGGATATCTT
GACTGATTTTTCCATGGAGGGCACAGTTAAGCCGCTAAAGGCATTATCCGCCAAGTA
CAATTTTTTACTCTTCGAAGACAGAAAATTTGCTGACATTGGTAATACAGTCAAATTGC
AGTACTCTGCGGGTGTATACAGAATAGCAGAATGGGCAGACATTACGAATGCACACG
GTGTGGTGGGCCCAGGTATTGTTAGCGGTTTGAAGCAGGCGGCGGAAGAAGTAACA
AAGGAACCTAGAGGCCTTTTGATGTTAGCAGAATTGTCATGCAAGGGCTCCCTAGCTA
CTGGAGAATATACTAAGGGTACTGTTGACATTGCGAAGAGCGACAAAGATTTTGTTAT
CGGCTTTATTGCTCAAAGAGACATGGGTGGAAGAGATGAAGGTTACGATTGGTTGATT
ATGACACCCGGTGTGGGTTTAGATGACAAGGGAGACGCATTGGGTCAACAGTATAGA
ACCGTGGATGATGTGGTCTCTACAGGATCTGACATTATTATTGTTGGAAGAGGACTAT
TTGCAAAGGGAAGGGATGCTAAGGTAGAGGGTGAACGTTACAGAAAAGCAGGCTGG
GAAGCATATTTGAGAAGATGCGGCCAGCAAAACTAAggcgcgcc**

Supplementary Tables

Supplementary Table 1. Plasmids used for strain construction. Promoters are represented by "pr", terminators are represented by "t".

Plasmid	Backbone	Insert	Source
pDB58	pKERG105	*ACT1*pr-VPEL222-*CYC1*t	this study
pDB60	pFA6-*his3MX6*	5xBS-*CYC180*pr-Kozak-mKate2-*ADH1*t	this study
pDB67	pFA6-*his3MX6*	1xBS-*CYC180*pr-Kozak-mKate2-*ADH1*t	this study
pDB72	pFA6-*his3MX6*	5xBS-*GAL1*pr-Kozak-mKate2-*ADH1*t	this study
pDB99	pFA6-*his3MX6*	2xBS-*CYC180*pr-Kozak-mKate2-*ADH1*t	this study
pDB100	pFA6-*his3MX6*	3xBS-*CYC180*pr-Kozak-mKate2-*ADH1*t	this study
pDB101	pFA6-*his3MX6*	4xBS-*CYC180*pr-Kozak-mKate2-*ADH1*t	this study
pDB102	pFA6-*his3MX6*	6xBS-*CYC180*pr-Kozak-mKate2-*ADH1*t	this study
pDB103	pFA6-*his3MX6*	*TDH3*pr-Kozak-mKate2-*ADH1*t	this study
pDB107	pFA6-*his3MX6*	5xBS-*SPO13*pr-Kozak-mKate2-*ADH1*t	this study
pDB110	pFA6-*his3MX6*	5xBS-*CYC180*pr-Kozak-Citrine-*ADH1*t	this study
pDB113	pKERG105	*ACT1*pr-mCitrine-VPEL222-*CYC1*term	this study
pDB78	pFA6-*his3MX6*	5xBS-*CYC180*pr-Kozak-mKate2-24xPP7SL-*ADH1*t	this study
pDB116	pRG215	*ACT1*pr-VPEL222-*CYC1*t	this study
pDB131	pRG215	*ACT1*pr-mCitrine-VPEL222-*CYC1*t	this study
pDB150	pFA6-*his3MX6*	5xBS-*CYC180*pr-Kozak-mCitrine-ADH1tail-*ADH1*t	this study
pDB152	pFA6-*his3MX6*	5xBS-*GAL1*pr-Kozak-mCitrine-URA3-*CYC1*t	this study

Supplementary Table 2. Strains used in this study. Promoters are represented by “pr”, terminators are represented by “t”.

Name	Genotype	Source	Data shown in Figure
BY4741	MATa *his3Δ1 leu2Δ0 met15Δ0 ura3Δ0*	Euroscarf	1d; S1a
BY4742	MATalpha *his3Δ1 leu2Δ0 lys2Δ0 ura3Δ0*	Euroscarf	-
DBY41	BY4741, *LEU2*::*ACT1*pr-VPEL222-*CYC1*t(pDB57)	this work	1d; S1a; S5
DBY42	BY4742, *LEU2*::*ACT1*pr-VPEL222-*CYC1*t(pDB57)	this work	-
DBY43	DBY41, *his3Δ*::5xBS-*CYC180*pr-Kozak-mKate2-*ADH1*t-*HIS3MX*(pDB60)	this work	1d,g; 2b-d; 3a; 4e S1a; S5, S7; S8; S9; S11;S12
DBY44	DBY41, *his3Δ*::5xBS-*GAL1*pr-Kozak-mKate2-*ADH1*t-*HIS3MX*(pDB72)	this work	2a; S5; S8
DBY45	DBY41, *his3Δ*::1xBS-*CYC180*pr-Kozak-mKate2-*ADH1*t-*HIS3MX*(pDB60)	this work	2a; S5; S8
DBY69	DBY41, *his3Δ*::2xBS-*CYC180*pr-Kozak-mKate2-*ADH1*t-*HIS3MX*(pDB99)	this work	2a-d; S5; S6;S7;S8;S11
DBY70	DBY41, *his3Δ*::3xBS-*CYC180*pr-Kozak-mKate2-*ADH1*t-*HIS3MX*(pDB100)	this work	2a; S5
DBY71	DBY41, *his3Δ*::4xBS-*CYC180*pr-Kozak-mKate2-*ADH1*t-*HIS3MX*(pDB101)	this work	2a; S5
DBY72	DBY41, *his3Δ*::6xBS-*CYC180*pr-Kozak-mKate2-*ADH1*t-*HIS3MX*(pDB102)	this work	2a; S5
DBY122	DBY41, *his3Δ*::5xBS-*SPO13*pr-Kozak-mKate2 -*ADH1*t-*HIS3MX*(pDB107)	this work	2a; S5;S8
DBY88	DBY41, *his3Δ*::5xBS-*CYC180*pr-Kozak-mKate2-24xPP7SL-*ADH1*t-*HIS3MX*(pDB78)	this work	S3
DBY73	BY4741, *his3Δ*::5xBS-*CYC180*pr-Kozak-mKate2-*ADH1*t-*HIS3MX*(pDB60)	this work	1d
DBY100	DBY41, *his3Δ*::*TDH3*pr-Kozak-mKate2-*HIS3MX*(pDB103)	this work	S1c; S9a,b
DBY104	DBY42, *his3Δ*::5xBS-*CYC180*pr-Kozak-Citrine-*ADH1*t-*HIS3MX*(pDB110)	this work	-
DBY105	DBY73, *LEU2*::*ACT1*pr-mCitrine-VPEL222-*CYC1*t(pDB113)	this work	3g; S11
DBY110	MATa/MATalpha, DBY43/DBY104	this work	3b,c; 4b,c; S10
DBY112	DBY73, *ACT1*pr-VPEL222-*CYC1*t (pDB116)	this work	3h; S12
DBY118	MATa/MATalpha, DBY69/DBY104	this work	S8a
DBY128	DBY73, *ACT1*pr-mCitrine-VPEL222-*CYC1*t (pDB131)	this work	S12a
DBY148	DBY41, 5xBS-*GAL1*pr-Kozak-mCitrine-*URA3*-*CYC1*t (pDB152)	this work	-
DBY149	DBY41, 5xBS-*CYC180*pr-Kozak-mCitrine-*ADH*tail-*ADH1*t (pDB150)	this work	1g, S4, S13e
DBY158	MATa/MATalpha, DBY148/BY4742	this work	5a, S14
DBY150	BY4741, *CAR1*::*CAR1*-mCitrine-*HIS3MX*	this work	S13a
DBY152	BY4741, *TDA1*::*TDA1*-mCitrine-*HIS3MX*	this work	S13a
DBY153	BY4741, *TMA108*::*TMA108*-mCitrine-*HIS3MX*	this work	S13a
DBY154	BY4741, *GPX2*::*GPX2*-mCitrine-*HIS3MX*	this work	S13a
DBY155	BY4741, *HOG1*::*HOG1*-mCitrine-*HIS3MX*	this work	S13a
DBY156	BY4741, *GLY1*::*GLY1*-mCitrine-*HIS3MX*	this work	S13a
DBY157	BY4741, *SOD1*::*SOD1*-mCitrine-*HIS3MX*	this work	S13a
DBY159	BY4741, *GCD11*::*GCD11*-mCitrine-*HIS3MX*	this work	S13a
DBY160	BY4741, *NIP1*::*NIP1*-mCitrine-*HIS3MX*	this work	S13a
DBY161	BY4741, *HEM15*::*HEM15*-mCitrine-*HIS3MX*	this work	S13a

Supplementary Table 3. Primers and smFISH probes used in this study. For the HIS3-integration and mCitrine-tagging primers, lowercase bases are complementary to plasmid sequences and uppercase bases are complementary to the yeast genome. All smFISH probes are labeled with CY3 at the 5' end. Probe sequences were obtained from ref. 31.

Primer / probe name	Sequence
HIS3_insertion_fwd	TCTTGGCCTCCTCTAGTACACTCTATATTTTTTTATGCCTCGGTAATGAgaaaccattattatcatgacattaacc
HIS3_insertion_rv	TATGGCAACCGCAAGAGCCTTGAACGCACTCTCACTACGGatcgatgaattcgagctcg
DB464_CAR1_fwd	TCTCTGCAGGTTGCGCCATTGCAAGGTGTGCATTGGGTGAAACCTTATTGggtgacggtgctggttta
DB465_CAR1_rv	CTAAAATAAAAAGAGAATGCTTATTTTGATAAAAGGGATGATGATATAAAatcgatgaattcgagctcg
DB468_TDA1_fwd	TAGGTGATGACGATAATGAGGACAGTATGGAAATTGATGATGACCTAGATggtgacggtgctggttta
DB469_TDA1_rv	TATATTACTGATTCTTGTTCGAAAGTTTTTAAAAATCACACTATATTAAAatcgatgaattcgagctcg
DB470_TMA108_fwd	CATTCCAGGTGAATGTTGATGATTCTGATGGTGATGAAACCATGCAGATAggtgacggtgctggttta
DB471_TMA108_rv	CCCAGGTAAAAAAAAAAAGAAAGAAAAAAAAGGTGAATTATCATTCAACAatcgatgaattcgagctcg
DB472_GPX2_fwd	AAGATGAAGAGTCCAGAAAGACTATAAGAAGAGACTTGGCCTCCTTGGTCggtgacggtgctggttta
DB473_GPX2_rv	GAAGACTACTAAATCTACGTACGATAGTGTGTTGAAATACTTGTAAAAAAatcgatgaattcgagctcg
DB474_HOG1_fwd	TAACAAAACCATCGTCCTTGGACCAAGAAATCCAAAGCCTGTTAAGTAAAggtgacggtgctggttta
DB475_HOG1_rv	AAATGTATTATTTATTGTTCCGGATAATTGCTTGATTATAGACAAAGATAatcgatgaattcgagctcg
DB484_SOD1_fwd	CTGGTAATGCCGGTCCAAGACCAGCCTGTGGTGTCATTGGTCTAACCAACggtgacggtgctggttta
DB485_SOD1_rv	CTTACTACTTACTTACATACGGTTTTTATTCAAGTATATTATCATTAACAatcgatgaattcgagctcg
DB486_GLY1_fwd	AGGTCGACGTTGATGGCAACGCTATCCGCGAAATAAAAACCTACAAATACggtgacggtgctggttta
DB487_GLY1_rv	CAATCCTAAAACAAAAACCCTAACAATACACATGATGCAACTGGAACGCAatcgatgaattcgagctcg
DB490_NIP1_fwd	TTACGGACTACGGTAACCAGGCCATACAGTACGCTAATGAGTTCCAACAGggtgacggtgctggttta
DB491_NIP1_rv	ACATCAAAAAGAAGTAAGAATGAGTGGTTAGGGACATTAAAAAAACACGTatcgatgaattcgagctcg
DB492_HEM15_fwd	ATGATCCTGTAAAGGACCTTTCATTGGTATTTGGCAATCACGAATCTACTggtgacggtgctggttta
DB493_HEM15_rv	ATAAATAAGAGAATATACTGATATTGAGATTGTGGGATGAATGGCCCTTAatcgatgaattcgagctcg
DB494_GCD11_fwd	TGATTGGTTGGGCAACCATTAAAAAGGGTACTACATTGGAACCCATCGCTggtgacggtgctggttta
DB495_GCD11_rv	TTTCAGATTAATTATGAAATTTTTGTCTTTGCAGTGGTTTTATTGGTTCCatcgatgaattcgagctcg
PP7 probe 1	5'-[Cyanine3]TTCTAGGCAATTAGGTACCTTA-3'
PP7 probe 2	5'-[Cyanine3]TTTCTAGAGTCGACCTGCAG-3'
PP7 probe 3	5'-[Cyanine3]AATGAACCCGGGAATACTGCAG-3'

Supplementary Table 4. Estimated parameters for the VP-EL222 model.

Parameter	Description	value: 5xBS-C180pr	value: 2xBS-C180pr
TF_{total} (molecules)	total cellular TF	2000	2000
k_{on} (min^{-1} * $(uW\ cm^{-2})^{-1}$)	light dependant VP-EL222 activation rate	0.0016399	0.0016399
k_{off} (min^{-1})	VP-EL222 dark-state reversion rate	0.34393	0.34393
k_{basal} (mRNA * min^{-1})	basal transcription rate	0.02612	0.24358
k_{max} (mRNA * min^{-1})	maximal induced transcription rate	13.588	11.031
K_d (molecules)	TF_{on} level required for achieving k_{max} / 2	956.75	1462.5
n (-)	hill coefficient	4.203	4.6403
k_{degR} (min^{-1})	mRNA degradation rate	0.042116	0.042116
k_{trans} (proteins * min^{-1} * $mRNA^{-1}$)	translation rate	1.4514	1.4514
k_{degP} (min^{-1})	protein degradation rate	0.007	0.007

Supplementary Table 5. Proteins used for calibration of protein copy numbers. The reporter molecule numbers represent the mean classified as being measured in minimal media and mid-log growth phase. See Supplementary Note 4 for further details on calibration.

Protein Name	Molecules per cell
Tda1p	5528
Tma108p	5909
Hog1p	6795
Gpx2p	7588
Hem15p	8271
Gcd11p	25373
Nip1p	30394
Car1p	36627
Sod1p	76172
Gly1p	104465

Supplementary Table 6. Parameters used for stochastic simulations of the VP-EL222 model. For the transcription-translation model (Supplementary Fig. 13c,d), multiple parameter combinations of k_{max} and k_{trans} were used (all are summarized in the table).

Parameter	Description	Two-state model (diploid/haploid)	Transcription-Translation
kTFtc(mRNA * min-1)	TF mRNA transcription rate	0.332 / 0.166	0.332
kdegTFr(min^{-1})	TF mRNA degradation rate	0.063	0.063
kTFtl(proteins * min^{-1}* $mRNA^{-1}$)	TF translation rate	32.6359	32.6359
kdegTFr(min^{-1})	TF degradation rate	0.007	0.007
kon(min^{-1} * (uW $cm^{-2})^{-1}$)	light dependant VP-EL222 activation rate	0.0016399	0.0016399
koff(min^{-1})	VP-EL222 dark-state reversion rate	0.34393	0.34393
kPon(min^{-1})	maximal Promoter on switch rate	1	-
kPoff(min^{-1})	maximal Promoter off switch rate	1	-
kbasal(mRNA * min^{-1})	basal transcription rate	0.02612	0.02612 / 0.002612 / 0.002612
kmax(mRNA * min^{-1})	maximal induced transcription rate	13.588	13.588 / 1.3588 / 0.13588
Kd(molecules)	TFon level required for achieving kmax/2	11756 / 5878	11756
n (-)	hill coefficient	4.203	4.6403
kdegR(min^{-1})	mRNA degradation rate	0.042116	0.042116
ktrans(proteins * min^{-1}* $mRNA^{-1}$)	translation rate	11.4639	11.4639 / 114.639 / 1146.39
kdegP(min^{-1})	protein degradation rate	0.007 (0.03 in Fig. S13e)	0.007

Supplementary Table 7. Parameters for hill function fit describing the mapping of Ura3p levels to cell growth (shown in Fig. 5). Equation: Growth-rate = k_m * $Ura3p^n$ / ($Ura3p^n$ + $K_d{}^n$), where Ura3p is equated to the measured mCitrine fluorescence.

Parameter	AM	PWM
k_m (h^{-1})	0.467	0.426
n	2.28	3.91
K_d	0.0186	0.0149

Supplementary References

1. Gordon, A. *et al.* Single-cell quantification of molecules and rates using open-source microscope-based cytometry. *Nat. Methods* **4,** 175–181 (2007).

2. Gnügge, R., Liphardt, T. & Rudolf, F. A shuttle vector series for precise genetic engineering of Saccharomyces cerevisiae. *Yeast* **33,** 83–98 (2016).

3. Hansen, A. S. & O'Shea, E. K. Promoter decoding of transcription factor dynamics involves a trade-off between noise and control of gene expression. *Mol. Syst. Biol.* **9,** 704 (2013).

4. Larson, D. R., Zenklusen, D., Wu, B., Chao, J. A. & Singer, R. H. Real-time observation of transcription initiation and elongation on an endogenous yeast gene. *Science* **332,** 475–478 (2011).

5. Belle, A., Tanay, A., Bitincka, L., Shamir, R. & O'Shea, E. K. Quantification of protein half-lives in the budding yeast proteome. *Proc. Natl. Acad. Sci. U. S. A.* **103,** 13004–13009 (2006).

6. Wang, Y. *et al.* Precision and functional specificity in mRNA decay. *Proc. Natl. Acad. Sci. U. S. A.* **99,** 5860–5865 (2002).

7. Sun, M. *et al.* Global analysis of eukaryotic mRNA degradation reveals Xrn1-dependent buffering of transcript levels. *Mol. Cell* **52,** 52–62 (2013).

8. Zoltowski, B. D., Nash, A. I. & Gardner, K. H. Variations in Protein–Flavin Hydrogen Bonding in a Light, Oxygen, Voltage Domain Produce Non-Arrhenius Kinetics of Adduct Decay. *Biochemistry* **50,** 8771–8779 (2011).

9. Motta-Mena, L. B. *et al.* An optogenetic gene expression system with rapid activation and deactivation kinetics. *Nat. Chem. Biol.* **10,** 196–202 (2014).

10. Eden, E. *et al.* Proteome half-life dynamics in living human cells. *Science* **331,** 764–768 (2011).

11. Schwanhäusser, B. *et al.* Global quantification of mammalian gene expression control. *Nature* **473,** 337–342 (2011).

12. Volfson, D. *et al.* Origins of extrinsic variability in eukaryotic gene expression. *Nature* **439,** 861–864 (2005).

13. Zopf, C. J., Quinn, K., Zeidman, J. & Maheshri, N. Cell-cycle dependence of transcription dominates noise in gene expression. *PLoS Comput. Biol.* **9,** e1003161 (2013).

14. Spencer, S. L., Gaudet, S., Albeck, J. G., Burke, J. M. & Sorger, P. K. Non-genetic origins of cell-to-cell variability in TRAIL-induced apoptosis. *Nature* **459,** 428–432 (2009).

15. Zechner, C., Unger, M., Pelet, S., Peter, M. & Koeppl, H. Scalable inference of heterogeneous reaction kinetics from pooled single-cell recordings. *Nat. Methods* **11,** 197–202 (2014).

16. Ho, B., Baryshnikova, A. & Brown, G. W. Unification of Protein Abundance Datasets Yields a Quantitative Saccharomyces cerevisiae Proteome. *Cell Syst* **6,** 192–205.e3 (2018).

17. Zenklusen, D., Larson, D. R. & Singer, R. H. Single-RNA counting reveals alternative modes of gene expression in yeast. *Nat. Struct. Mol. Biol.* **15,** 1263–1271 (2008).

18. Curran, K. A., Karim, A. S., Gupta, A. & Alper, H. S. Use of expression-enhancing terminators in Saccharomyces cerevisiae to increase mRNA half-life and improve gene expression control for metabolic engineering applications. *Metab. Eng.* **19,** 88–97 (2013).

19. Thattai, M. & van Oudenaarden, A. Intrinsic noise in gene regulatory networks. *Proceedings of the National Academy of Sciences* **98,** 8614–8619 (2001).

20. Rullan, M., Benzinger, D., Schmidt, G. W., Milias-Argeitis, A. & Khammash, M. An Optogenetic Platform for Real-Time, Single-Cell Interrogation of Stochastic Transcriptional Regulation. *Mol. Cell* **70,** 745–756.e6 (2018).

21. Raser, J. M. & O'Shea, E. K. Control of Stochasticity in Eukaryotic Gene Expression. *Science* **304,** 1811–1814 (2004).

22. Elowitz, M. B., Arnold, J. V., Siggia, E. D. & Swain, P. S. Stochastic Gene Expression in a Single Cell. *Science* **297,** 1183–1186 (2002).

23. Gillespie, D. T. Exact stochastic simulation of coupled chemical reactions. *J. Phys. Chem.* **81,** 2340–2361 (1977).

24. Griesbeck, O., Baird, G. S., Campbell, R. E., Zacharias, D. A. & Tsien, R. Y. Reducing the Environmental Sensitivity of Yellow Fluorescent Protein. *J. Biol. Chem.* **276,** 29188–29194 (2001).

25. Shcherbo, D. *et al.* Far-red fluorescent tags for protein imaging in living tissues. *Biochem. J* **418,** 567–574 (2009).

26. Brachmann, C. B. *et al.* Designer deletion strains derived from Saccharomyces cerevisiae S288C: a useful set of strains and plasmids for PCR-mediated gene disruption and other applications. *Yeast* **14,** 115–132 (1998).

27. Longtine, M. S. *et al.* Additional modules for versatile and economical PCR-based gene deletion and modification in Saccharomyces cerevisiae. *Yeast* **14,** 953–961 (1998).

28. McIsaac, R. S. *et al.* Synthetic gene expression perturbation systems with rapid, tunable, single-gene specificity in yeast. *Nucleic Acids Res.* **41,** e57 (2013).

29. Vidal, M., Brachmann, R. K., Fattaey, A., Harlow, E. & Boeke, J. D. Reverse two-hybrid and one-hybrid systems to detect dissociation of protein-protein and DNA-protein interactions. *Proc. Natl. Acad. Sci. U. S. A.* **93,** 10315–10320 (1996).

30. Hocine, S., Raymond, P., Zenklusen, D., Chao, J. A. & Singer, R. H. Single-molecule analysis of gene expression using two-color RNA labeling in live yeast. *Nat. Methods* **10,** 119–121 (2013).

31. Ochiai, H., Sugawara, T., Sakuma, T. & Yamamoto, T. Stochastic promoter activation affects Nanog expression variability in mouse embryonic stem cells. *Sci. Rep.* **4,** 7125 (2014).
